# 2D Materials and Heterostructures at Extreme Pressure

**DOI:** 10.1002/advs.202002697

**Published:** 2020-11-10

**Authors:** Linglong Zhang, Yilin Tang, Ahmed Raza Khan, Md Mehedi Hasan, Ping Wang, Han Yan, Tanju Yildirim, Juan Felipe Torres, Guru Prakash Neupane, Yupeng Zhang, Quan Li, Yuerui Lu

**Affiliations:** ^1^ Institute of Microscale Optoelectronics College of Physics and Optoelectronic Engineering Shenzhen University Shenzhen 518060 China; ^2^ Research School of Electrical, Energy and Materials Engineering College of Engineering and Computer Science The Australian National University Canberra ACT 2601 Australia; ^3^ International Center for Computational Methods and Software College of Physics Jilin University Changchun 130012 China

**Keywords:** 2D materials, diamond anvil cell (DAC), high pressure, metallization, optoelectronics, superconducting

## Abstract

2D materials possess wide‐tuning properties ranging from semiconducting and metallization to superconducting, etc., which are determined by their structure, empowering them to be appealing in optoelectronic and photovoltaic applications. Pressure is an effective and clean tool that allows modifications of the electronic structure, crystal structure, morphologies, and compositions of 2D materials through van der Waals (vdW) interaction engineering. This enables an insightful understanding of the variable vdW interaction induced structural changes, structure–property relations as well as contributes to the versatile implications of 2D materials. Here, the recent progress of high‐pressure research toward 2D materials and heterostructures, involving graphene, boron nitride, transition metal dichalcogenides, 2D perovskites, black phosphorene, MXene, and covalent–organic frameworks, using diamond anvil cell is summarized. A detailed analysis of pressurized structure, phonon dynamics, superconducting, metallization, doping together with optical property is performed. Further, the pressure‐induced optimized properties and potential applications as well as the vision of engineering the vdW interactions in heterostructures are highlighted. Finally, conclusions and outlook are presented on the way forward.

## Graphical abstract

Pressure can modify the electronic structures, crystal structures, morphologies, and compositions of 2D materials, which contributes to their versatile implications. Here, the recent progress of high‐pressure research of 2D materials and associated heterostructures is summarized. The structure, phonon dynamics, superconducting, metallization, doping, optical property, optimized properties, and future research directions of them are discussed in detail.

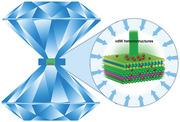

## 1 Introduction

After the successful exfoliation of graphene, the investigations into 2D materials have undergone a burgeoning growth. This is ascribed to their intrinsic material properties, highly tunable electronic and optoelectronic properties, as well as potential technological applications, such as field‐effect transistors (FET), optoelectronic devices, photovoltaic devices, topological insulators and electrocatalysts, etc.^[^ ^1^, ^2^, ^3^, ^4^, ^5^, ^6^, ^7^ ^]^ 2D materials are normally formed by loosen van der Waals (vdW) bonds, where the external stimuli (e.g., vdW interaction engineering) can spark many extraordinary electronic or optoelectronic properties (e.g., insulating, semiconducting, metallization, superconducting, interesting light–matter interactions, etc.).^[^ ^8^, ^9^, ^10^ ^]^ Many strategies including pressure, reducing dimension, intercalation, fabricating heterostructures, chemical doping, alloying, electrical gating, etc., have been used to modify the fixed properties of 2D materials and extend their applications.^[^ ^11^, ^12^, ^13^ ^]^ Pressure, being an important thermodynamic variable, could provide a powerful method to tune the atomic, electronic, and crystal structures of 2D materials without introducing damages and impurities.^[^ ^14^, ^15^, ^16^ ^]^ As an example, the pressure‐induced dramatic changes in electronic structures and structural phase transitions of transition metal dichalcogenides (TMDs),^[^ ^17^, ^18^, ^19^, ^20^ ^]^ boron nitride (BN),^[^ ^21^, ^22^, ^23^, ^24^, ^25^, ^26^ ^]^ MXene,^[^ ^27^, ^28^, ^29^ ^]^ and black phosphorene (BP)^[^ ^30^, ^31^, ^32^, ^33^, ^34^ ^]^ have been reported.^[^ ^15^, ^17^, ^18^, ^19^, ^20^, ^35^, ^36^, ^37^ ^]^ Compared with their bulk counterparts, 2D TMDs exhibit thickness‐dependent phase transitions (from 2H_c_ to 2H_a_) under high pressure, and thinner films are more sensitive to pressure in terms of Raman vibration modes.^[^ ^38^ ^]^ Another example is the occurrence of amorphizations of 2D perovskites and covalent–organic frameworks (COFs) due to high pressure, which leads to significant changes of their optoelectronic properties.^[^ ^15^, ^39^, ^40^, ^41^, ^42^, ^43^, ^44^ ^]^ Moreover, charge transfer and doping effects under high pressure are observed in 2D materials and associated heterostructures.^[^ ^37^, ^45^, ^46^ ^]^ To date, there have been few reviews about how pressure influences vdW interactions, electronic and optoelectronic properties of 2D materials. Therefore, systematic discussions about the pressurized changes of vdW interactions, structures, and properties are imperative to enable the versatility of 2D materials and associated heterostructures.

In this review, we firstly introduce the structure and properties of 2D materials under ambient pressure (Section 2). In the case of TMDs, the common structures and associated properties are reviewed in terms of different phases. Then, the working principle of diamond anvil cell (DAC) is discussed in detail (Section 3). Favor from commons and underlying physics, pressurized structure, phonon dynamics, metallization, superconducting, doping, optical property, and optimized optoelectronic properties as well as potential applications are summarized (Section 4). Furthermore, we vision the vdW interaction engineering of heterostructures in terms of vibration, exciton modulation, charge transfer, and Moiré pattern (Section 5). Finally, the challenges and future opportunities for high‐pressure research of 2D materials and correlated heterostructures are discussed.

## 2 Ambient‐Pressure Structure and Properties

The structure and corresponding optoelectronic properties of 2D materials have been well summarized by other reviews.^[^ ^47^, ^48^, ^49^ ^]^ To distinguish our work from others, we only discuss some fundamentals that are correlated with high‐pressure research, including crystal structure, electronic structure, bandgap changes and structural phase transitions, phonon properties, etc.

Since graphene was exfoliated from graphite in 2004, 2D materials research has experienced an upsurge.^[^ ^49^ ^]^ Graphene comprises of single‐atom‐thick carbon atoms with honeycomb lattices, showing unique electronic and optoelectronic properties.^[^ ^1^, ^2^, ^3^, ^4^, ^5^, ^6^, ^7^, ^49^, ^50^, ^51^ ^]^ Due to a two‐carbon unit cell, graphene has an electronic structure with a point in which two bands touch (Dirac point). The energy dispersion shows a linear dependence near the Dirac point and electrons act like Dirac fermions having a speed of 1/300 light speed at the Dirac point. They lead to many intriguing electronic/optoelectronic properties such as high charge carrier mobility, ambipolar field effect, anomalous quantum Hall effect, ballistic transport, chirality, Klein paradox, and so on. Extremely high charge mobility of 200 000 cm^2^ V^−1^ S^−1^ has been reported in suspended graphene, showing promising potentials in ultrafast electronics and optoelectronics.^[^ ^52^ ^]^ For ambipolar field‐effect transport, it provokes graphene‐based devices for electronics. Also, the feature of two carbon atoms per unit cell causes graphene's chirality. Compared with the carriers in other materials (metals or semiconductors), graphene possess a relative nature of Dirac fermions that enables carriers to cross barriers differently (Klein paradox). Meanwhile, attributed to its super high charge mobility, an observation of the quantum Hall effect has been reported in graphene at room temperature.^[^ ^53^ ^]^ Besides that, its unique crystal structure exhibits intriguing optical properties. It is found that the fine structure constant of graphene determines its optical transparency. For white light, graphene shows an absorption value of 2.3%, whereas it exhibits 2–3% light absorption in the ultraviolet to infrared (IR) range, which is highly related to its electronic structure.^[^ ^54^ ^]^ Accounting for its ultrathin thickness, the absorption of graphene is quite attractive. Moreover, graphene has an ultralow reflectance (e.g., 0.1% for monolayer (1L) and 2% for 10 layers). Monolayer and bilayer (2L) graphene show a robust and layer‐dependent interband optical transition, which can be modulated by electrical gates. Additionally, their different infrared optical responses have been demonstrated, where monolayer graphene does not have an apparent feature in the normalized change of IR reflectivity and bilayer indicates a prominent peak at ≈350 meV. However, graphene does not have a bandgap which hampers its optoelectronic applications demanding high on/off switching ratios.^[^ ^15^ ^]^ To address the gapless issue of graphene, a lot of strategies have been proposed including chemical functionalizations,^[^ ^55^ ^]^ stress, high electrical field and nanoribbons,^[^ ^56^, ^57^ ^]^ etc., which more or less sacrifices its high mobility. Therefore, exploring more alternative materials has become a hot topic.^[^ ^58^ ^]^

**Figure 1:**
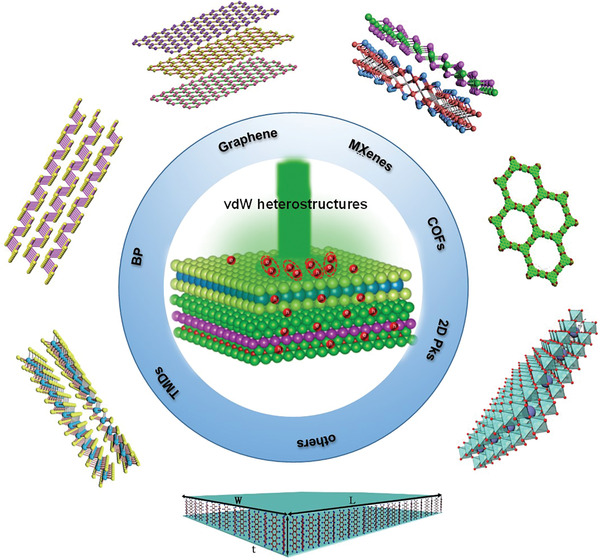
Schematic illustration of several common 2D materials.

**Figure 2:**
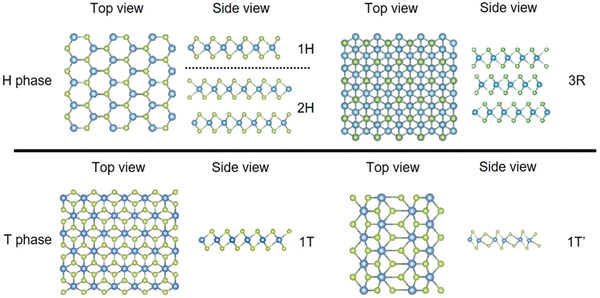
Common crystal structures of various TMD phases. Reproduced with permission.^[^ ^55^ ^]^ Copyright 2020, Annual Reviews, Inc.

In the past decade, a majority of 2D ultrathin materials involving transition metal dichalcogenides (TMDs, e.g., MoS_2_, MoSe_2_, WSe_2_, WS_2_, TiS_2_, TaS_2_, etc.),^[^ ^49^, ^59^, ^60^, ^61^ ^]^ BN,^[^ ^21^, ^22^, ^23^, ^24^, ^25^, ^26^, ^35^, ^62^, ^63^, ^64^, ^65^, ^66^, ^67^, ^68^, ^69^, ^70^, ^71^ ^]^ BP,^[^ ^72^, ^73^, ^74^, ^75^, ^76^, ^77^, ^78^, ^79^, ^80^, ^81^, ^82^, ^83^, ^84^ ^]^ 2D organic crystals (e.g., 2D small molecular and polymers),^[^ ^85^, ^86^, ^87^, ^88^, ^89^, ^90^, ^91^, ^92^, ^93^, ^94^, ^95^ ^]^ 2D perovskites,^[^ ^96^, ^97^, ^98^, ^99^, ^100^, ^101^, ^102^ ^]^ 2D covalent–organic frameworks,^[^ ^58^, ^86^, ^103^, ^104^, ^105^, ^106^, ^107^, ^108^, ^109^, ^110^, ^111^ ^]^ 2D MXenes,^[^ ^15^, ^112^, ^113^, ^114^, ^115^ ^]^ etc., have been studied (**Figure** **1**). They exhibit excellent optical, electronic, and mechanical properties, as well as thermal conductivity. In the case of TMDs, they connect through weak vdW bonds and show layer‐dependent bandgaps and high mobility, which are highly desired in the applications of electronic and optoelectronic devices. They contain a layer of transition metal atom (e.g., Mo, W, etc.) and two layers of chalcogen atoms (e.g., S, Se, Te, etc.), and the former is sandwiched by the latter. They are usually grouped as hexagonal (1H/2H), trigonal (3R), tetragonal (1T), and monoclinic (1T′), in which 1, 2, or 3 represents the layer number per unit in the direction of the *z* or *c*‐axis (i.e., the direction perpendicular to the (0001) plane) (**Figure** **2**).^[^ ^55^ ^]^ These different crystal structures correspond to the divergent optoelectronic properties of 2D materials. For instance, MoS_2_ exhibits semiconducting properties in the 1H/2H phases, whereas it is metallic in the 1T phase.^[^ ^14^, ^15^ ^]^ The majority of TMDs are indirect semiconductors in bulk form, whereas they manifest direct‐gap semiconductors at a monolayer, which has been substantiated by many characterizations through spectroscopic tools.^[^ ^116^, ^117^, ^118^, ^119^ ^]^ Attributed to phonon‐assisted process and negligible quantum yield, bulk 2H‐MoS_2_ has a quite weak photoluminescence (PL), while the monolayer shows a significant enhancement in PL that indicates a direct‐gap semiconductor.^[^ ^118^, ^119^ ^]^ The lack of weak interlayer coupling for monolayers is responsible for this indirect–direct transition, which has been substantiated by interlayer thermal expansion in few‐layer MoSe_2_.^[^ ^120^ ^]^ The direct bandgap transition enables its implications in high‐performance optoelectronic devices (transistors with super high on/off ratio at room temperature).^[^ ^121^ ^]^ Additionally, the observation of two peaks (A and B) in the absorption spectra for 1L and 2L MoS_2_ has been demonstrated, which are assigned to transitions between split valence bands (VBs) and conduction bands (CBs). For 1L MoS_2_, the energy splitting of *A* to *B* merely depends on spin–orbit coupling (SOC) and it originates from interlayer coupling and SOC for bulk counterparts. The new and well‐controlled degree of freedom beyond charge and spin, originating from coupled spin and valley physics, results in valleytronics. At the energy levels of bandgap regions, a bounded state‐exciton (an electron–hole pair) forms through Coulomb interactions. Due to 2D features, strong spatial confinement and decreased screening effect of 2D materials enhance many‐body interactions and form robust quasiparticles, for example, trions, biexcitons, and other multiple‐exciton complexes, which are extremely important for optoelectronic applications such as light‐emitting diodes, lasers, optical modulators, etc.^[^ ^83^ ^]^ By using density functional theory (DFT), the phonon dispersions of 1L TMDs are revealed. It is composed of the three acoustic and six optical branches that come from the nine vibration modes at the Γ point. Here, the three acoustic branches are classified by in‐plane longitudinal acoustic, transverse acoustic, and out‐of‐plane acoustic modes. Among them, the first two modes have a linear dispersion and higher frequency as compared to the third one. Correspondingly, the six optical branches include two in‐plane longitudinal optical, two in‐plane transverse optical, and two out‐of‐plane optical branches. Owing to TMDs being polar materials, longitudinal optical‐transverse optical splitting is observed in infrared active phonon modes. It is explained by the coupling between lattice and the macroscopic electric field. The latter is formed by the relative displacement of metal and chalcogen atoms at the long‐wavelength range. Also, TMDs have a bandgap between acoustic and optical branches (MoS_2_ and WS_2_: ≈100 cm^−1^; WSe_2_: ≈30 cm^−1^; MoSe_2_: ≈15 cm^−1^). The external stimulus could effectively engineer the electric, phonon, thermal, and mechanical properties of 2D TMDs. A strain‐tuned bandgap of 1L and 2L MoS_2_ has been reported.^[^ ^122^, ^123^ ^]^ The theoretical calculations reveal that the biaxial‐strain engineered bandgap is more efficient than that of uniaxial strain.^[^ ^124^ ^]^ With the applied external electrical fields, the neutral and charged excitons in 1L and 2L TMDs can be heavily modulated.^[^ ^76^, ^125^, ^126^, ^127^ ^]^ In the case of monolayer MoTe_2_, both negatively charged and positively charged excitons have been observed in gate‐tuned PL measurements, where their PL intensity increases with the increase of doping level and it shows binding energies of around 24 and 27 meV, respectively.^[^ ^125^ ^]^ Moreover, PL peaks of 1L and 2L MoS_2_ show a strong dependence with temperature.^[^ ^127^ ^]^ According to theoretical calculations, it was found that the band structure of 2L MoS_2_ evolves from indirect at room temperature to direct at low temperature, whereas monolayers have an opposite tendency. These different evolutions dominate the carrier relaxation pathways within PL process, resulting in a faster increase in PL intensity for bilayers as compared with monolayers while decreasing temperature. More interestingly, by low‐temperature PL measurements, the electrical tuning of K–K direct PL transitions in 2L MoS_2_ has been observed, which facilitates the studies of exciton and trion dynamics.^[^ ^127^ ^]^ The investigations of 2D TMDs under external stimulus provoke their versatile applications in future electronic and optoelectronic devices.^[^ ^51^, ^83^, ^125^, ^128^, ^129^, ^130^ ^]^ Due to the graphene‐level high mobility and tunable bandgap (0.3 eV (bulk) to 2.0 eV (monolayer)^[^ ^31^, ^73^ ^]^),^[^ ^72^, ^73^, ^74^, ^76^, ^77^, ^78^, ^79^, ^80^, ^81^, ^82^ ^]^ black phosphorous has attracted widespread attention. Although BP has narrow bandgaps that variate from mid‐infrared to near‐infrared wavelengths, it overcomes the drawbacks of gapless‐graphene and relatively large‐bandgap TMD semiconductors.^[^ ^126^, ^131^ ^]^ BP has an orthorhombic structure and one layer contains two special puckered atomic layers. From the top view, it exhibits a distorted hexagonal structure, where each phosphorous atom connects with three nearby phosphorous atoms via the corresponding lengths (2.244 and 2.224 Å) and angles (96.34° and 102.09°). It implies that BP has two distinct in‐plane directions (i.e., the armchair direction having puckered structure along *x* and the zigzag direction having ridge structure along *y*).^[^ ^132^ ^]^ The calculated electronic band structure of BP with different thickness demonstrates a different trend with that of TMDs. It always has a direct‐bandgap against any thickness. But its CB and VB touch at the *Z* point in bulk BP, whereas they meet at the Γ point in mono/few‐layer BP. Notably, for monolayer BP, its valence band top is quite flat. Although its valence band maximum (VBM) probably deviates from the Γ point, monolayer BP is thought as a direct bandgap semiconductor due to the small separation (<10 meV) between VBM and Γ point. Recently, the bandgap of BP covering the visible‐to‐IR spectral range (0.3 to 2.0 eV) has been substantiated by scanning tunneling microscopy,^[^ ^133^ ^]^ infrared relative extinction spectra,^[^ ^128^ ^]^ and theoretical calculations.^[^ ^72^ ^]^ Due to a highly tunable direct bandgap, the spectral range of its optical response is significantly expanded. Additionally, its strong exciton effects demonstrate a promising potential for light emission. Recently, various local geometry tunings including tensile, compressive strain, and curvature, have been applied to engineer the bandgap of BP, which even leads to a phase transition of metal‐semiconductor. For monolayer BP, owing to the puckered orthorhombic lattice structure combing with the D_2h_ symmetry, it shows strong in‐plane anisotropy. Its carrier effect mass in the zigzag direction is ≈10‐fold larger as compared with the armchair direction. In terms of optics, the optical selection rules dominate its anisotropy and optical transitions are highly sensitive to the momentum operator p̂_x/y_. In monolayer BP, the finite *p̂_y_* matrix element permits electronic transitions, whereas it has prohibited transitions with *y*‐polarization due to the zero *p̂_y_* matrix element. This robust anisotropy not only can be used to determine the crystallographic axes but can also be applied in light generation, manipulation, and detection. Moreover, oxidation has been utilized to modify optoelectronic properties, where oxygen chemisorption/physisorption is used as defect sources in the bandgap states. Therefore, by controlling the degree of oxidation, it is possible to tune the bandgap of BP, enabling the implementation of optoelectronics.^[^ ^72^ ^]^ Despite its multiple merits, its wide applications remain challenging due to the instability of 2D BP.^[^ ^126^, ^131^ ^]^

Recently, 2D organic–inorganic hybrid perovskites joined the 2D material family, having the strong potential for low‐cost and high‐performance photovoltaic and optoelectronic devices.^[^ ^101^ ^]^ They also show some intriguing properties such as intrinsic ambipolar transport, high optical absorption coefficient in the visible spectra range, high quantum efficiency, and a long carrier diffusion length.^[^ ^96^, ^101^, ^134^, ^135^, ^136^, ^137^ ^]^ These 2D organic–inorganic hybrid perovskites could be described by a formula of (RNH_3_)_2_(CH_3_NH_3_)*_m_*_−1_A*_m_*X_3_ *_m_*_+1_, in which R, A, and X represent the alkyl or aromatic moiety, metal cation, and halide, respectively; *m* represents the layer number of the metal cation and this layer is sandwiched by two layers of organic chains.^[^ ^100^, ^138^, ^139^ ^]^ In contrast with other 2D materials, they possess a more flexible and deformable tetragonal or orthorhombic structure. This results in anomalous structural relaxations and bandgap changes.^[^ ^100^ ^]^ As reported, the optical and electrical properties of hybrid perovskites could be tuned by modifying their thickness or the proportions of the two halogens. Liu et al. reported the synthesis of 2D CH_3_NH_3_PbI_3_ perovskite crystals where the PL is tunable with the evolution of layer number and composition.^[^ ^101^ ^]^ Applied high‐quality 2D perovskites, a high‐efficiency photodetector has been demonstrated, exhibiting an increased current under both 405 and 532 nm laser irradiation. At 1 V, the corresponding photoresponsivity was 22 and 12 A W^−1^, respectively.^[^ ^101^ ^]^ Moreover, it is found that the electronic structures of hybrid perovskites generally show similar characteristics. The VBM is comprised of an *n*p^6^ orbital from the halogen (*n* represents the principal quantum number and it is 3, 4, and 5 for Cl, Br, and I, respectively) and *n*s^2^ from the metal (*n* is 4, 5, and 6 for Ge, Sn, and Pb, respectively). Mostly, the conduction band minimum (CBM) is composed of the empty *np* ^0^ orbitals originating from the metal. The organic cations play a role in the lattice constant that impacts band structures. Nevertheless, the symmetry of perovskite structures dominates their corresponding band structure. As an example, a wider electronic band structure is observed in the cubic structure with a smaller effective mass and higher mobility, which demonstrates the huge potential to apply cubic perovskites to technological applications.^[^ ^16^ ^]^ Also, halide perovskites usually have direct bandgap and high optical coefficients. By modifying the chemical compositions (e.g., the ratio of constituent halides), the bandgap of perovskites can be modulated surpassing several hundred nanometers. These highly tunable properties provide a new route toward engineering the light absorption in optoelectronic and photovoltaic devices as well as the open‐circuit in solar cells. However, the properties of ultrathin 2D hybrid perovskites, such as poor chemical stability, fast crystallization rate, and intrinsically non‐van der Waals‐type 2D features, setup barriers for practical applications. Therefore, it is imperative to reinforce an understanding of 2D materials’ structure–property relationships to explore the broader implications of 2D materials.^[^ ^14^, ^126^ ^]^

## 3 High‐Pressure Technique: DAC

Compared to other methods such as temperature, uniaxial and biaxial strain, DAC is a powerful tool to produce ultrahigh static pressure,^[^ ^14^ ^]^ which could tune the electronic and optoelectronic properties of 2D materials through engineering their vdW interactions, bond lengths, angles, and electronic state energies.^[^ ^140^, ^141^ ^]^ In the following section, we introduce the components, working principle, and corresponding synchrotron characterization tools of DAC.

**Figure 3:**
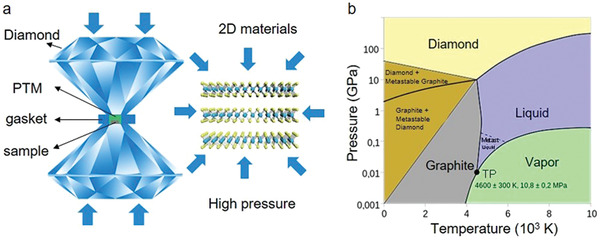
High‐pressure technique. a) Schematic of common diamond anvil cell (DAC). b) Phase diagram of carbon. Reproduced with permission.^[^ ^142^ ^]^ Copyright 2020, Wikimedia Foundation.

DAC is comprised of two diamond anvils, one gasket and a sample chamber (**Figure** **3**a).^[^ ^47^ ^]^ Diamond acts as a chemically inert substrate, which not only has ultrahigh hardness but also an ultrahigh phase‐transition temperature (i.e., 4200 K and 10 GPa) that is difficult to reach (Figure 3b).^[^ ^142^ ^]^ Moreover, it is transparent for wavelengths from infrared to X‐rays and gamma rays, having low luminescence and high light transmission efficiency. Given these, DAC emerges as a popular testing platform for high‐pressure and relevant experiments.^[^ ^142^ ^]^ For the gasket, it contains the designated sample, ruby, and pressure transmitter media (PTM) (Figure 3a),^[^ ^48^ ^]^ among which PTM is critical to generate a uniform compression on the designated sample. They could be classified as: 1) liquid soft solids (e.g., NaCl and BN); 2) solvents (e.g., silicone oil, alcohols, fluorine); 3) noble gases (e.g., Ne, He, and Xe). The above three PTM produce a quasi‐hydrostatic pressure, hydrostatic pressure, and above hydrostatic pressure, respectively.^[^ ^47^ ^]^ Besides, PTM influences the strain level, pressure anisotropy, and gradients. Therefore, they could decide the reproducibility of experimental results.^[^ ^16^, ^143^, ^144^, ^145^ ^]^ More importantly, to prevent PTM from reacting with the 2D materials, they are normally chemically inert.^[^ ^47^ ^]^

In the DAC experiments, by using two opposing diamonds to squeeze the 2D materials, a high static pressure (i.e., above 100 GPa) could be obtained.^[^ ^140^ ^]^ In particular, the pressure could be described as *P* = *F/A*, where *P*, *F*, and *A* represent the applied pressure, force, and contact area of force, respectively. According to this equation, the strategy of applying a small force on an ultrasmall area is preferred to obtain a high pressure.^[^ ^142^ ^]^ Moreover, considering the pressure value of the Earth center (≈360 GPa), the available high pressure (≈300 GPa) from DAC, allows for structure–property relations to be explored under high pressure.^[^ ^140^, ^146^ ^]^ Furthermore, a standard pressure‐dependent ruby fluorescence technique that employs the *R*_1_ fluorescence from small ruby pieces are used to monitor and calibrate the pressure.^[^ ^48^ ^]^

Furthermore, integrations of multiple synchrotron techniques with DAC have promoted investigations of fundamental physics and materials science of 2D materials. They include X‐ray diffraction (XRD) characterizing long‐range crystal structures, the pair distribution function (PDF) revealing short‐range local bonding features at the atomic scale, X‐ray Raman spectroscopy (XRS) showing the chemical bonding changes, X‐ray spectroscopy surveying the phonon density of states (DOS), and X‐ray imaging demonstrating the dynamic process and internal strain.^[^ ^47^ ^]^ These combinations have boosted the studies of the vdW interactions, structures, optoelectronic and electronic properties of 2D materials, enhancing the understanding of the novel phenomena and fundamental physics under pressure.^[^ ^16^ ^]^

## 4 Optoelectronic and Physical Properties Evolutions

To enable versatile applications in nano‐optoelectronic devices and circuits, it is imperative to break the fixed properties of 2D materials. Here, we systematically discuss the pressure‐induced evolutions of material properties through engineering vdW interactions, including structural tuning (Section 4.1), phonon dynamics (Section 4.2), metallization (Section 4.3), superconducting (Section 4.4), doping (Section 4.5), and optical property tuning (Section 4.6). Meanwhile, the optimized optoelectronic properties and potential applications are concluded (Section 4.7).

### 4.1 Tuning of Structure

**Figure 4:**
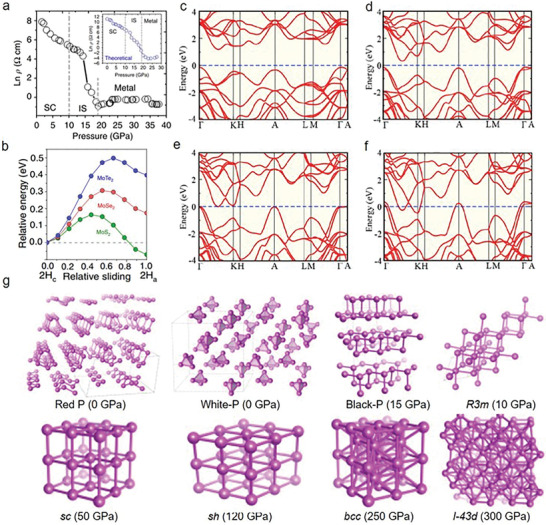
Tuning of structures under high pressure. a) Resistivity as a function of pressure in MoS_2_. b) The relative energy of MoTe_2_, MoSe_2_, and MoS_2_ as a function of relative sliding ranging from 2H_c_ to 2H_a_. c–f) Theoretical band structure as a function of pressure under ambient pressure, 23 GPa, 41 GPa, and 58 GPa, respectively. g) Crystal structures from various allotropes of phosphorus under different pressure. a) Reproduced with permission.^[^ ^19^ ^]^ Copyright 2014, Springer Nature. b–f) Reproduced with permission.^[^ ^20^ ^]^ Copyright 2015, Springer Nature. g) Reproduced with permission.^[^ ^32^ ^]^ Copyright 2017, American Physical Society.

As mentioned, 2D materials’ structures determine their physics, electronic and optoelectronic properties, dominating their potential applications.^[^ ^14^, ^15^ ^]^ To date, plenty of studies on the structural and property changes of 2D materials through engineering vdW interactions have been reported.^[^ ^14^, ^15^, ^16^, ^48^, ^147^ ^]^ Nayak et al. demonstrated the pressure‐dependent electronic, vibrational, optical, and structural properties of multilayered MoS_2_ (**Figure** **4**a). Structural lattice distortions and subsequent electronic transitions from semiconducting to the metallic state are disclosed at ≈19 GPa through ab initio calculations.^[^ ^19^ ^]^ Moreover, single‐crystal XRD results in MoS_2_ have been demonstrated over a wide pressure–temperature (*P*–*T*) domain, in which an isosymmetric 2H_c_ to 2H_a_ polytype phase transition has been investigated, conjecturing a charge density wave (CDW) before superconductivity at 25 GPa. In contrast to powder, the transition pressure range of TMD single crystals has a significant decrease, implying a probable coupling with the deviatoric stresses. The XRD measurements presented the structural information of 2H_a_‐MoS_2_ under high pressure which does not have structural distortion or broken symmetry. This relates to CDW in the broad *P*–*T* range that approaches 78 GPa and ranges from 20 to 298 K. The electrical conductivity and unusual Raman features can be ascribed to a purely electronic transformation (e.g., Lifshitz transition and the occurrence of the long excitonic insulator state). Importantly, the distinct features of MoS_2_ as compared with TiSe_2_, NbSe_2_, and TaS_2_ can be ascribed to the different number of valence electrons, which is included for establishing the electronic bands around the Fermi level.^[^ ^148^ ^]^ Besides that, a high pressure induced novel superstructure phase in 1T‐VSe_2_ has been demonstrated, exhibiting changes from weak vdW bonding to Heisenberg covalent bonding.^[^ ^149^ ^]^ Unexpectedly, the experimental results show the emergence of the novel superstructure at 15.5 GPa that is not suppressed as normal. This is caused by Fermi surface nesting that is strengthened by distortions coming from high pressure. It is also found that the superstructure can occur in both 2D structures and pressure engineered 3D structures.^[^ ^149^ ^]^ Furthermore, the structural, vibrational, and topological properties of topological insulator Bi_1.5_Sb_0.5_Te_1.8_Se_1.2_ under high pressure have been investigated. The observation of two structural phase transitions has been reported, where the rhombohedral R$\bar{3}$m‐monoclinic C2/m phase transition occurs at 13 GPa and a disordered I4/mmm phase starts at around 22 GPa. Interestingly, within the R$\bar{3}$m phase, the alloy experiences multiple electronic transitions such as the bandgap transition from indirect to direct at around 5.8 GPa, a bulk bandgap closing accompanying by the occurrence of a Dirac semimetal state at around 8.2 GPa, and a semimetal state at 12.1 GPa. The electron–phonon coupling contributes to transitions, which is supported by anomalous *c*/*a* ratio as well as consistency of full width at half maximum (FWHM) and Dirac semimetal phase. In contrast with other binary end members including Bi_2_Te_3_, Bi_2_Se_3_, and Sb_2_Te_3_, Bi_1.5_Sb_0.5_Te_1.8_Se_1.2_ demonstrated higher pressure value of the structural phase transitions and anomalies.^[^ ^150^ ^]^ Also, a phase transition from the orthorhombic T_d_ to a new T′ phase in Tungsten ditelluride (WTe_2_) has been demonstrated experimentally and theoretically.^[^ ^151^ ^]^ In particular, WTe_2_ indicates a strong plane‐parallel/plane‐vertical vibrational anisotropy attributed to its Raman tensor. While increasing the pressure, Raman peaks at around 120 cm^−1^ show a redshift that hints the evolution of a T_d_ phase. Due to the occurrence of inversion symmetry, the Weyl states disappear at 8 GPa where a phase transition of Td‐T′ happens.^[^ ^151^ ^]^ Zhao et al. reported a transform from a 2D layered network to a 3D structure in MoSe_2_, which is free of structural transitions.^[^ ^20^ ^]^ Moreover, the layer sliding simulations demonstrate that MoS_2_ has a lower energy barrier maximum (0.15 eV) compared with MoTe_2_ and MoSe_2_ (Figure 4b), which explains why a 2H_a_ structure of MoS_2_ is more observable. Also, the ab initio calculations provide the electrical band structures at ambient pressure, 23, 41, and 58 GPa, respectively, whose variations unveil the bandgap narrowing effects and metallization transitions with the increase of pressure (Figure 4c–f).^[^ ^20^ ^]^ In contrast with TMDs, due to different crystal structures at ambient pressure, one predicts distinct structure transitions and properties for black phosphorus under high pressure. The pressure‐induced electronic topological and structural transitions have been investigated for black phosphorus experimentally and theoretically.^[^ ^33^ ^]^ The accurate HSE calculations demonstrated the occurrence of band inversion at 1.2 GPa. The strong topological index *ν*_0_ = 0 for *P* < 1.2 GPa and *ν*_0_ = 1 for *P* ≥ 1.2 GPa are observed, confirming the transition of the topological insulator phase at above 1.2 GPa.^[^ ^33^ ^]^ Furthermore, the crystal structures’ evolutions of phosphorus are demonstrated under different pressures (Figure 4g).^[^ ^32^ ^]^ Under ambient pressure, red phosphorus was stable with triclinic structure (P‐I), whereas BP (Cmca) nearly degenerated. While increasing the pressure from 3 to 16 GPa, A7‐R3m phase (P‐II) emerges. Continuing to increase pressure to 120 GPa, the simple cubic (sc) structure (P‐III) becomes dominant. As the pressure ramps up to ≈225 GPa, a simple hexagonal (sh) structure appears. Intriguingly, a stable crystal structure‐bcc (I‐43d) was observed from 250 to above 350 GPa. Also, pressure‐induced structural transformations of BN (e.g., from hexagonal boron nitride (h‐BN) to a hexagonal close‐packed wurtzite structure (w‐BN)) have been investigated theoretically and experimentally.^[^ ^22^, ^24^, ^25^, ^26^, ^64^, ^65^ ^]^ Segura et al. reported the observation of a nonreversible phase transition from hexagonal BN to wurtzite at 13 GPa, which has been substantiated by infrared reflectance, transmission, and Raman measurements.^[^ ^22^, ^24^ ^]^ Meng et al. demonstrated the formation of sp^3^ bonding in compressed BN, revealing the structural transformation mechanism.^[^ ^25^ ^]^ In detail, with the increase of pressure, the electronic structure of B and N evolve and consequently, structural transitions happen. This can be explained by a direct bonding mechanism, where sp^3^ bonds simultaneously establish along the *a* and *c*‐axis directions of the hexagonal structure, forming a 3D tetrahedron framework. High‐pressure‐induced evolutions of B and N bonding together with interesting commons and differences provide a new platform to investigate pressurized phenomena such as polymerization, metallization, superconductivity, semiconductivity, etc.^[^ ^25^ ^]^ Moreover, a shear‐induced phase transition from disordered nanocrystalline h‐BN to w‐BN has been detected at room temperature under 6.7 GPa after applying large plastic shear in a rotational DAC (RDAC). Intriguingly, similar structural transformations were not observed under pressure up to 52.8 GPa. This is ascribed to the transition of h‐BN to a disordered phase with closed‐packed buckled layers that occurs at the initial stage of both high‐pressure cases. In contrast, under shear, an irreversible transition to w‐BN occurs, whereas another transition is reversible under hydrostatic compression. One representative explanation is that the transformation process highly depends on the dislocations of plastic flow in grains, where the nucleation process happens at high pressure together with the deviatoric stress concentrator at strain‐induced defect tips. In terms of little grains, extra plastic flow exists at grain boundaries due to the atomic realignments in localized shear regions. This functions the same with thermal activation under high pressure, resulting in the structural transformation to w‐BN.^[^ ^26^ ^]^ Pressure‐induced structure changes provide a new pathway to develop novel 2D material devices with desired electronic, optoelectronic, and structural properties.^[^ ^32^ ^]^

### 4.2 Phonon Dynamics

**Figure 5:**
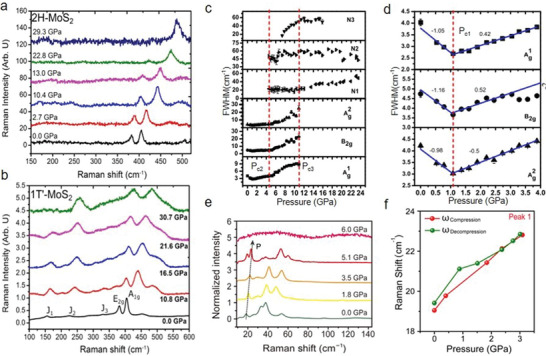
Phonon dynamics under high pressure. a,b) Raman spectra of monolayer 2H‐MoS_2_ and 1T′ MoS_2_ under the indicated pressure. It shows the pressure‐dependent phonon dynamics of different‐phase MoS_2_ monolayer. c) FWHM evolutions as a function of pressure from BP. The vertical dashed lines represent the pressure points of the structural phase transitions. d) FWHM evolutions of BP under pressure ranging from 0 to 4 GPa. e) Raman spectra of 2D perovskite (PEA)_2_PbI_4_ under the indicated pressure. f) Raman peak position evolution of Peak 1 during the compression and decompression. a,b) Reproduced with permission.^[^ ^18^ ^]^ Copyright 2014, American Chemical Society. c,d) Reproduced with permission.^[^ ^33^ ^]^ Copyright 2017, American Physical Society. e,f) Reproduced with permission.^[^ ^39^ ^]^ Copyright 2017, American Association for the Advancement of Science.

Raman spectroscopy is a sensitive tool to probe the phonon dynamics and investigate 2D materials’ lattice variations under high pressure.^[^ ^17^, ^18^, ^19^, ^20^, ^35^, ^37^ ^]^ By analyzing Raman active optical phonon modes, the corresponding doping level, layer number, atomic structure, crystal structure, composition, the physical and chemical properties of 2D materials can be determined.^[^ ^17^, ^18^, ^19^, ^20^, ^31^, ^32^, ^33^, ^35^, ^39^, ^152^ ^]^ As an example, the high‐pressure properties of unsupported vertical graphene nanosheets (VGNs) have been investigated by in situ Raman spectroscopy.^[^ ^153^ ^]^ It demonstrates the relationship between pressure (*P*) and G‐mode frequency (*ω*) for unsupported VGNs up to 40 GPa. After 16 GPa, a disturbance of the long‐range order in graphene has been observed, verified by *ω* having a discontinuous curve against *P*. This leads to a deviation away from the 2D layer structure. According to the apparent G‐band up to 40 GPa, it was found that local sp^2^ hybridization persists at these high pressures. Also, VGNs illustrate the healable ability and high stability in decompression.^[^ ^153^ ^]^ Moreover, an interesting observation of pressure‐induced thermal properties of 2D silicon nanosheets has been reported by characterizing phonon dynamics.^[^ ^154^ ^]^ First, their structural phase transitions are found highly related to the size and shape of silicon 2D nanosheets by studying the high‐pressure properties up to 21 GPa through synchrotron X‐ray powder diffraction. While increasing the size from 9.3 to 15.2 nm, the irreversible I (cubic)–II (tetragonal)–V (hexagonal) phase transitions occur, whereas an X‐ray amorphous phase pops up within decompression below 8 GPa. The experimental results disclose that plenty of 1D nanowires (aspect ratios > 10) formed via growing twinned as well as along 〈111〉 direction. Also, a transition from 2D morphology to 1D nanowire occurs, endowing a diamond structure. The molecular dynamic simulations illustrate that the thermal conductivity shows a declining tendency with the decrease of dimensionality. More importantly, the decrease of dimensionality can improve ZT coefficient (ZT = *S* ^2^ *Tσ*/*κ*, where *S* is the Seebeck coefficient, *k* is thermal conductivity, *σ* is electrical conductivity at specific temperature *T*). These findings demonstrate that pressure‐driven silicon nanomaterials or nanowires have significant potentials in high‐performance thermoelectrics.^[^ ^154^ ^]^ Additionally, the increase of thermal conductivity for MoS_2_ under high pressure has been reported.^[^ ^155^ ^]^ Through picosecond transient thermoreflectance characterization, bulk MoS_2_ shows an increasing cross‐pane thermal conductivity from 3.5 to around 25 W m^−1^ K^−1^ under about 9% cross‐plane compressive strain from DAC. This dramatic evolution, coming from strain‐induced stronger interlayer interactions, significantly tuned phonon dispersions and decreasing phonon lifetime attributed to the unbundling effect along the cross‐plane direction, which has been illustrated via theoretical calculations and coherent phonon spectroscopy measurements. Notably, the changeable electronic thermal conductivity plays a neglected role in pressurized properties.^[^ ^155^ ^]^ On the other hand, the optical phonon vibration modes of Mo_0.5_W_0.5_S_2_ have been investigated up to 40 GPa.^[^ ^156^ ^]^ While increasing pressure, the two in‐plane E_2g_ and disorder‐activated longitudinal acoustic phonon modes show a hardened and suppressed effect by Raman characterizations. Two A_1g_ modes of ternary compounds, similar to that of pristine MoS_2_ and WS_2_, exhibit the same Raman shift tendency as pristine compounds while increasing pressure. Differently, under high pressure, a new Raman peak of 470 cm^−1^ occurs in the ternary compounds, which is the disorder‐activated pressure‐induced out‐of‐plane Raman mode. As pressure increases over 30 GPa, a Raman peak of 340 cm^−1^ occurs, which represents the additional disorder‐activated vibration mode. These results reveal the strengthened interlayer interactions in ternary compounds, deepening the understanding of the electronic, optical, and structural properties under extreme conditions.^[^ ^156^ ^]^ Also, the lattice vibrations of 2H‐MoS_2_ monolayer have been characterized by Raman spectroscopy, which discloses an apparent evolution of lattice under high pressure (**Figure** **5**a).^[^ ^18^ ^]^ It was observed that the in‐plane Raman mode (E_2g_) begins to decrease after surpassing 16 GPa. This is because the compressive strain becomes dominant under large hydrostatic pressures, limiting the movement of E_2g_. In contrast, the out‐of‐plane vibration mode (A_1g_) still protrudes even at 30 GPa. The different increasing rates between A_1g_ and E_2g_ can be attributed to distinct vibration types. A_1g_ modes represent the transverse vibrations of S—S atom, whereas E_2g_ modes are the longitudinal vibrations of Mo and S atoms in the opposite directions. The evolution of Mo—S bond length decides the features of E_2g_ modes. With the increase of the hydrostatic pressure, A_1g_ mode compressions are more preferred than that of E_2g_, implying that the transverse vibrations of S—S atom move faster compared with the in‐plane moment of Mo—S atom. Consequently, this leads to a higher increasing rate of A_1g_ modes in contrast with E_2g_ modes. Moreover, a stable metallic state 1T′‐MoS_2_ with pressure has been discovered, where corresponding J_2_ and A_1g_ and E_2g_ modes appear to be dominant at high pressure.^[^ ^18^ ^]^ These three active phonon modes (i.e., 150 cm^−1^ (J_1_), 225 cm^−1^ (J_2_), 325 cm^−1^ (J_3_), at the ambient pressure) commonly occur in 1T′‐MoS_2_ instead of 2H‐MoS_2_ (Figure 5a,b). With the increase of pressure, J_3_ vanishes from 10 GPa, whereas the corresponding J_2_ and J_1_ show an increasing tendency. The former supports the coalescence of J_3_ and E_2g_ modes at high pressure.^[^ ^18^, ^157^ ^]^ They further verified the merging using theoretical calculations where they investigated how pressure influences the vibration properties of 1T′‐MoS_2_. While increasing pressure, the compression of the lattice in the out‐of‐plane direction (0.08 Å GPa^−1^) is higher than that of the in‐plane (0.03 Å GPa^−1^). This reveals that the S—S atom moves much faster than that of Mo—S, which results in a slow increase in E_2g_ (1.3 cm^−1^ GPa^−1^) as compared with the A_1g_ (2.5 cm^−1^ GPa^−1^) mode.^[^ ^23^ ^]^ In contrast with other TMDs, the A_1g_ shift of monolayer MoS_2_ is much larger and exhibits a stronger response with pressure.^[^ ^158^ ^]^ Compared with their bulk counterparts, the reason for a clear deviation is the lack of interlayer interactions along the out‐of‐plane axis direction in monolayer MoS_2_. Also, there is no intermediate state between semiconducting and metallic state, evidenced through the metallization or structural transitions of monolayer MoS_2_ that is not found even at above 30 GPa.^[^ ^18^ ^]^ These above phonon hardening effects of E_2g_ and A_1g_ modes together with the suppression of E_2g_ modes have been observed in multilayer WS_2_ and these could be extended to other TMDs.^[^ ^17^ ^]^ The suppression effect correlates tightly with the broadening of full width at half maximum at higher pressure. Moreover, for WS_2_, the intensity ratio of A_1g_ to E_2g_ ramps up with pressure. At a high‐pressure range, the A_1g_ mode becomes more notable with the complete disappearance of the E_2g_ mode.^[^ ^17^ ^]^ The apparent phonon hardening effects are ascribed to the anisotropic compression in the different directions (i.e., the out‐of‐plane and in‐plane directions) and enhanced interlayer interactions induced by the increasing pressure.^[^ ^17^, ^18^, ^19^, ^20^, ^159^ ^]^ Monolayer WS_2_ under high pressure (up to around 25 GPa) and on different substrates including Si/SiO_2_ and DAC surfaces has been investigated.^[^ ^160^ ^]^ According to the occurrence of Raman‐inactive B modes, different‐degree structural distortions have been observed. Attributed to additional strain from decreasing volume in Si and corrugation of the SiO_2_ surface, a split of out‐of‐plane B and A_1_′ modes become notable.^[^ ^160^ ^]^ In the case of monolayer WSe_2_, Raman measurements demonstrate that the lattice disorder increased with the increase of pressure and this is proved by the enhanced intensity of one phonon mode LA(M). According to the investigation of lattice structure changes, the asymmetrical pressure is considered to be responsible for lattice distortions, leading to evolutions of the band structure. These findings provide important references for investigating the mechanical, electrical, and thermal conduction properties of 2D materials.^[^ ^161^ ^]^ Moreover, the pressure‐driven vibrational properties of 2D Janus S—W—Se and S—Mo—Se monolayers have been demonstrated. They show a very small response to pressure, which differs from traditional semiconductors. After forming a vibrational response, 2D Janus layers do not experience a phase transition under pressure up to 15 GPa. Their vibration modes are lack of monotonic response to pressure.^[^ ^162^ ^]^ Furthermore, pressure‐dependent phonon dynamics of black phosphorus are also discussed experimentally and theoretically (Figure 5c,d).^[^ ^33^ ^]^ It is concluded that: 1) FWHM of first‐order Raman modes attain a minimum at ≈1.1 GPa and this is associated with the anomalies of electron–phonon coupling at electronic topological transition; In detail, through first‐principle calculations, a phase transition from a semiconductor to topological insulator occurs at a low‐pressure range. The calculated values of Z_2_ topological invariants further verified the evolution of electronic topology that indicates the transformation from a band to a topological insulating state. 2) Unusual B_2g_ and $A_{g}^{2}$ modes appear at ≈7.4 GPa and new modes (N_1_, N_2_, and N_3_) manifest in the rhombohedral phase. The new features exhibit anomalous softening with the increase of pressure, due to the unusual structural evolutions.^[^ ^33^ ^]^ To explore the relationship between mode softening and structural evolutions, the internal parameter *d*_1_, *d*_2_, *α*_1_, and *α*_2_ were investigated. Here, *d*_1_ and *d*_2_ represent the bond length of in‐plane P atoms and the distance out‐of‐plane P atoms, respectively; *α*_1_ and *α*_2_ represent the bond angles of two *d*_1_ bonds and between *d*_1_ and *d*_2_ bonds, respectively. Since the atomic vibrations of $A_{g}^{1}$ modes include *d*_2_ bonds’ extension and *d*_2_ shows a monotonically decreasing tendency with the increase of pressure, $A_{g}^{1}$ turns into hardening. Correspondingly, the atomic vibrations of B_2g_ modes modify *α*_2_ which indicates a decreasing tendency with pressure. The extension of *d*_1_ bonds is included in the atomic displacement of $A_{g}^{2}$ modes. In particular, *d*_1_ declines while pressure increases to 6 GPa and then starts to increase within 7–11 GPa. Comparatively, the evolution of *d*_2_ is much larger as compared to that of *d*_1_ with the increase of pressure. This is also verified due to the larger softening magnitude of B_2g_ compared to the $A_{g}^{2}$ modes. Moreover, BP is an extremely soft material that shows a decrease of 24% in volume at 14.7 GPa compared to 0 GPa, which corresponds to the large softening of B_2g_ and $A_{g}^{2}$ modes with the increase of pressure. For the new modes, N_1_, N_2_, and N_3_ are assigned to $E_{g}^{2}$, A_1g_, and $E_{g}^{1}$, respectively, which demonstrate softening with the increase of pressure. According to theoretical calculations, A7 phase (hexagonal structure) is a stable state at pressures variating from 4 to 12 GPa, after this range unstable modes in phonon dispersion occur. Also, N_1_ and N_2_ are still observable up to 24 GPa (sc phase) and they are explained by the zone boundary acoustic modes in the sc phase. More recently, pressure‐stimulated phonon dynamics in 2D hybrid perovskites were investigated. The investigations demonstrate the presence of some notable vibration modes at low wave numbers (e.g., below 50 cm^−1^) and this plays a key role in molecular interactions and orientation of benzene rings (Figure 5e).^[^ ^39^, ^163^, ^164^ ^]^ Given this, the interactions of neighboring benzene rings distributed at different layers of (PEA)_2_PbI_4_ are described by low‐wave number vibration modes. An apparent blueshift with the increase of pressure is observed and this indicates the different increasing tendency of intermolecular (interlayer) vibration frequencies. This enhanced intermolecular interaction is ascribed to the compressed reduction of interlayer distance. At specific pressures (e.g., below 3.5 GPa), Raman shift shows reversible phenomenon (Figure 5f). As the pressure increases to 5.1 GPa, a split of Raman peaks occurs and then above 6 GPa Raman modes would disappear completely. Furthermore, the phonon dynamic characterizations of 2D COF were reported, revealing the incomplete amorphizations and collapse of crystal structures.^[^ ^44^ ^]^ These investigations about phonon dynamics not only unveil the variations of vdW interactions, structure, and property in 2D materials, but also enable an in‐depth understanding of their fundamental physics.

### 4.3 Metallization

**Figure 6:**
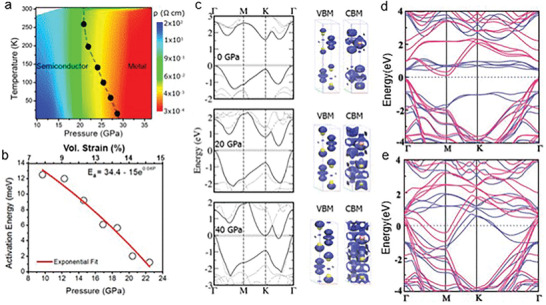
Pressure‐induced metallization. a) Temperature–pressure contour plot of resistivity under various pressure, showing the phase transition from semiconducting to the metallic state. b) Arrhenius curve demonstrating the emergence of the metallic phase at a pressure beyond 22 GPa. c) Calculated band structure at 0, 20.4, and 39.5 GPa, showing that the bandgap approaches zero with the increase of pressure. Moreover, a complete metallization was observed at 39.5 GPa. d,e) The calculated band structures of 2D VS_2_: under ambient pressure and 18.68 GPa. a–c) Reproduced with permission.^[^ ^17^ ^]^ Copyright 2015, American Chemical Society. d,e) Reproduced with permission.^[^ ^11^ ^]^ Copyright 2017, Wiley‐VCH.

While increasing pressure, the electronic structure, crystal structure, morphologies, and compositions of 2D materials could change, leading to the emergence of a metallic state or superconducting state.^[^ ^14^, ^165^, ^166^, ^167^ ^]^ For TMDs, when the interlayer spacing reduces with the increase of pressure, the enhanced vdW interactions of chalcogenide atoms stimulate the crossover of the valance band maxima and the conduction band minima over the Fermi level (*E*_F_).^[^ ^14^, ^17^, ^20^ ^]^ The metallization critical pressure is proportional to the layer number at the 2D limit.^[^ ^17^ ^]^ As a typical example, the corresponding transition from semiconductor to metal (S‐M) for WS_2_ occurs at ≈22 GPa (**Figure** **6**a).^[^ ^17^ ^]^ The electrical resistivity shows a decrease of six orders, whereas the carrier density exhibits a four‐order increase. Additionally, the minimum resistivity of ≈3 × 10^−4^ Ω cm at 36 GPa and ≈10^−4^ Ω cm at ambient pressure of the semimetallic or metallic TMDs are in the same order, confirming the metallization again. Moreover, an exponential decrease of the activation energy (*E*_a_) with pressure is observed in an Arrhenius plot and this supports that the metallic state emerges from ≈22 GPa (Figure 6b). In particular, while increasing the pressure, the bandgap becomes smaller and *E*_a_ approaches zero. As the pressure surpasses ≈22 GPa, the intrinsic band closure and 100% metallization take place (Figure 6c). Correspondingly, the energy level shift of VBM and CBM occurs because of the enhanced vdW interactions.^[^ ^17^, ^19^ ^]^ P_z_ orbital from S atoms and D_z2_ orbitals from W atoms constitute VBM and it moves upwards with the increase of pressure. The bonding P_z_ orbitals from S build up some part of CBM, whose downward shift originates from the reduction of interlayer distance as well as enhanced overlap (Figure 6c).^[^ ^17^, ^168^, ^169^, ^170^ ^]^ Interestingly, in contrast with multilayer MoS_2_, the metallization critical pressure for WS_2_ is higher, which is ascribed to the layer modifications of the electronic and transport properties.^[^ ^17^, ^19^ ^]^ Also, WS_2_ demonstrates a progressive drop in resistivity with pressure, which differs from the sudden drop of MoS_2_, revealing the slow tuning process of their electronic structure and bandgap.^[^ ^17^, ^19^ ^]^ More interestingly, in complete contrast with MoS_2_, multilayer molybdenum diselenide showed pressure‐induced metallization without structural transition.^[^ ^20^ ^]^ This stability of structures is due to the chalcogenide anions. But the electronic structure of MoSe_2_ is heavily engineered by high pressure in DAC, which modifies its optoelectronic properties. From the optical density (OD), a transfer from the semiconducting state to metallic state is distinguished. Applying the empirical model (i.e., in the case of indirect‐bandgap semiconductor, the absorption coefficient scales proportionally to the square of the energy difference between photon energy and bandgap),^[^ ^171^ ^]^ an *E*_g_ of ≈0.4 eV at 20.2 GPa is obtained. While increasing pressure from 0 to 35.1 GPa, *E*_g_ approaches zero. Qualitatively, the bandgap–pressure relationship could be described by a parabolic fit. Moreover, the electrical resistivity with the increase of pressure was measured as well, substantiating the metallization.^[^ ^20^ ^]^ At low pressures (below 23.4 GPa), a negative d*ρ*/d*T* at high temperatures is found, showing a semiconducting state. Then, from 27.0 to 37.0 GPa, a positive d*ρ*/d*T* appears, demonstrating the occurrence of a metallic state. As the pressure rises above 47 GPa, the metallization of MoSe_2_ throughout all temperatures is observed. Furthermore, Guo et al. demonstrated the vdW interactions play a dominant role in the electronic state of 2D VS_2_, which not only allows for the precise controllability of the electronic state but also avoids dramatic structural changes.^[^ ^11^ ^]^ The in situ temperature‐dependent resistance experiments disclose the transition from semiconductor to metal. In particular, 2D VS_2_ shows the semiconducting properties at low pressures, where a CDW transition occurs at ≈250 K and the corresponding transition temperature decreases with the increase of pressure. While increasing pressure, the CDW transition becomes weakened owing to the decreasing cell parameters.^[^ ^172^, ^173^ ^]^ The evolution of semiconductor to metal happens at ≈5 GPa, accompanying with the complete suppression of the CDW transition. In detail, one‐order higher conductivity is observed at high pressure as compared with that of ambient pressure, verifying the metallizations. Theoretical calculations were also conducted, revealing that the metallization transition originates from the d orbital states of vanadium. At ambient pressure, the valence band maximum locates at ≈0.3 eV below *E*_F_ (Figure 6d)*_._* The vanadium d orbitals decide the conduction band minima, whereas the valence band maxima are determined by the S p_x_ and p_y_ orbitals. While increasing pressure to 5.64 GPa, a balanced compression is reached in the VS_2_. The larger overlap of S p orbitals and dz^2^ is realized, due to the significant decrease of the cell parameters, which supports the wider dispersion and metallization. Continuously increasing pressure to 18.68 GPa, the density of states at *E*_F_ appears partly influenced by the in‐plane compression while the overlaps of d orbitals are enhanced by the axial compression. They are together with the DOS from the S p orbitals enhancement at *E*_F_ (Figure 6e)*_._* Given these, vanadium is the key to engineer the electronic state in pristine VS_2_. In a word, the metallization appears through engineering vdW interactions, which provides an effective route to modulate the electrical band structure, enabling the tuning of spin properties and the electronic state of VS_2_.^[^ ^11^, ^172^ ^]^ Until now, the state transitions from an insulator or semiconductor state to the metallic state in various materials involving CdI_2_,^[^ ^174^ ^]^ Bi_2_X_3_, Sb_2_X_3_, and Ag_2_X (e.g., X = S, Se, and Te), MOF,^[^ ^44^ ^]^ perovskites,^[^ ^39^, ^40^, ^41^, ^42^, ^43^ ^]^ BP,^[^ ^32^ ^]^ etc.,^[^ ^20^ ^]^ have been investigated. In these metallizations, the large structural reconstructions or atomic movements mostly take place to close the vdW gaps or lead to first‐order structural transitions although the unique metallization of hybrid perovskites without structural transition has been found.^[^ ^39^, ^42^, ^175^ ^]^ Furthermore, we summarize the critical pressure of metallization for various 2D materials in **Table** **1**. This impressive pressure‐induced metallization and associated optoelectronic properties further extend the potential applications of 2D materials and contribute to the investigation of their novel behaviors.

**Table 1:** Critical pressure of metallization or superconducting in common 2D materials

2D material		Metallization critical pressure [GPa]	Superconducting critical pressure [GPa]	Other phases
2D perovskite	(PEA)_2_PbI_4_	53.7 ± 8.2^[^ ^39^ ^]^		
	(CH_3_NH_3_)PbI_3_	Above 60 (RT)^[^ ^42^ ^]^		
	Cs_3_Bi_2_I_9_	28 (338 K)^[^ ^200^ ^]^		
2D TMDs	ZrTe_2_	T‐ZrTe_2_ ≈ 2 (H‐Zr‐Te_2_ ≈ 6)^[^ ^201^ ^]^		
	1T‐TiSe_2_		2–4 (1.8 K)^[^ ^191^ ^]^	
	WTe_2_		10.5 (2.8 K);^[^ ^183^ ^]^ 4–5 (300 K)^[^ ^184^ ^]^	
	WS_2_	22 (280 K)^[^ ^17^ ^]^		
	WSe_2_	51.7 (RT)^[^ ^202^ ^]^		
	ZrS_2_	5.6–25^[^ ^186^ ^]^	5.6–25 (1.1–1.9 K) or 25–100 (0.3–0.1 K)^[^ ^186^ ^]^	
	MoTe_2_	14.9^[^ ^203^ ^]^		
	MoS_2_	30 (270 K);^[^ ^167^ ^]^ 9 (RT)^[^ ^19^ ^]^	90 (3 K)^[^ ^167^ ^]^	Intermediate state: 10–19; semiconducting state: 0–10^[^ ^19^ ^]^
CdI_2_		62 (240 K)^[^ ^174^ ^]^		35 (270 K)^[^ ^174^ ^]^ semiconducting phase
FeCL_2_		47 (300 K)^[^ ^204^ ^]^		
NI_2_		19 (310 K)^[^ ^205^ ^]^		
MgC_2_			Ambient pressure (15 K)^[^ ^206^ ^]^	
BP		1.7, 10^[^ ^207^ ^]^	Above 5 (6–13 K);^[^ ^181^ ^]^ above 10 (5–10 K);^[^ ^182^ ^]^ 11–30 (4–10.7 K), 15 (6 K)^[^ ^207^ ^]^	
MoSe_2_		40.7–60^[^ ^20^ ^]^		0–40.7 semiconducting phase
CaC_2_			43 (7.9 K) and 95 (9.8 K)^[^ ^185^ ^]^	
CaC_6_			7.5 (15.1 K)^[^ ^208^ ^]^	

### 4.4 Superconducting

**Figure 7:**
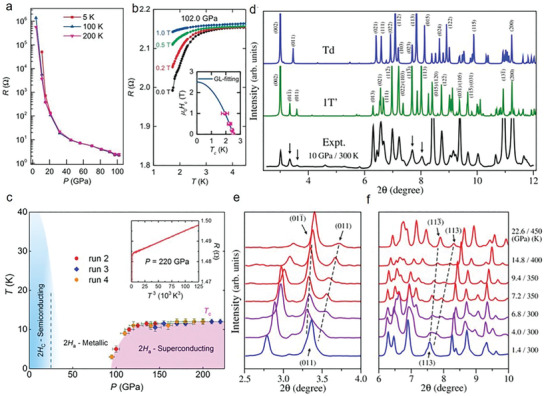
Pressure‐induced superconductivity. a) Pressure‐dependent isothermal resistance of ReS_2_ at 5, 100, and 200 K. b) Magnetic field‐dependent resistance at 102 GPa. The upper critical field *µ*_0_ *H*_c2_ is presented in the inset. The experimental data are fitted by the Ginzburg–Landau (GL) formula. c) Pressure–temperature (*P–T*) phase diagram of 2H‐MoS_2,_ demonstrating the respective regions of superconducting and metallic phases. d) Theoretical XRD spectra at 10 GPa together with experimental data at the indicated condition. e,f) Experimental XRD spectra showing clear splittings in the peaks of (011) and (113), demonstrating the occurrence of phase transitions. a,b) Reproduced with permission.^[^ ^167^ ^]^ Copyright 2017, Springer Nature. c) Reproduced with permission.^[^ ^113^ ^]^ Copyright 2018, American Physical Society. d–f) Reproduced with permission.^[^ ^184^ ^]^ Copyright 2016, American Physical Society.

Pressure can significantly modify 2D materials’ vdW interactions, crystals structure, and electronic structure and this provides unique opportunities to investigate their superconducting properties.^[^ ^176^, ^177^, ^178^, ^179^, ^180^ ^]^ Recently, pressure‐induced superconductivity has been reported in ReS_2_,^[^ ^180^ ^]^ 2H_a_‐MoS_2_,^[^ ^167^ ^]^ BP,^[^ ^181^, ^182^ ^]^ WTe_2_,^[^ ^183^, ^184^ ^]^ CaC_2_,^[^ ^185^ ^]^ ZrS_2_,^[^ ^186^ ^]^ etc. In the case of ReS_2_, a tetragonal *I*4_1_/amd structure establishes at around 90 GPa, showing superconducting capabilities.^[^ ^180^ ^]^ **Figure** **7**a presents the isothermal resistance with the increase of pressure, in which a clear drop of resistance at above 90 GPa is prevalent. To substantiate superconductivity, different external magnetic fields were also applied along the *c*‐axis of ReS_2_ at 102.0 GPa during the electrical resistance experiments (Figure 7b). It shows a slow‐down drop of resistance and a tendency toward a low‐temperature curve with the increase of magnetic fields, proving the emergence of the superconducting phase.^[^ ^180^ ^]^ Chi et al. also investigated the superconductivity in pristine 2H_a_‐MoS_2_ and this is explained by the occurrence of a new flat Fermi pocket at ultrahigh pressure.^[^ ^167^ ^]^ Using the external magnetic field, superconductivity was again verified. In particular, as the external magnetic field varies from 0 to 6.5 T at 220 GPa, the superconducting critical temperature (*T*_c_) progressively moves toward a lower value. This is common in the transition of bulk superconductivity. From the *P–T* diagram of 2H‐MoS_2_, the superconductivity transition occurs at around 90 GPa and it exhibits a constant *T*_c_ from ≈135 GPa (Figure 7c).^[^ ^167^, ^187^, ^188^, ^189^ ^]^ This exotic *T*_c_ (*P*) hints a band‐overlap metallization, where 2D electronic DOS at the Fermi level [*N*(*E*_F_)] remains invariant with the increase of carrier concentrations.^[^ ^190^ ^]^ The above phenomenon is quite similar to that of 1T‐TaS_2_^[^ ^173^ ^]^ and Bi_2_Se_3_,^[^ ^190^ ^]^ whereas it shows a large difference compared to 1T‐TiSe_2_^[^ ^191^ ^]^ and T_d_‐WTe_2_.^[^ ^167^, ^183^ ^]^ For instance, Bi_2_Se_3_’s superconductivity starts from 11 GPa. While increasing the pressure, its *T*_c_ nearly remains unchanged with the increase of carrier concentrations (one‐order enhancement). This was ascribed to the balance of the volume‐dependent *T*_c_ via the phonon cutoff frequency 〈*ω*_c_〉 and Fermi level [*N*(*E*_F_)]. It is evidenced by the Bardeen–Cooper–Schrieffer (BCS) relationship and the McMillan strong coupling formalism.^[^ ^192^, ^193^ ^]^ Moreover, it was observed that the superconductivity of 2H_a_‐MoS_2_ is an intrinsic property without structural phase transition, decomposition, and amorphization as characterized by synchrotron X‐ray diffraction.^[^ ^167^ ^]^ In detail, at ambient pressure, the lattice constants and Wyckoff position of S atoms are *a* = 3.1618 (1) Å, *c* = 12.3043(4) Å and 4f (1/3, 2/3, 0.622). The corresponding ones at 52 GPa are *a* = 3.1618 (1) Å, *c* = 12.3043(4) Å and 4f (1/3, 2/3, 0.622), respectively. This denotes that 2H_a_‐MoS_2_ is rather stable toward pressure, which has been further confirmed by Raman measurements at room temperature. Furthermore, density functional theory calculations present the relationship between the band structure and pressure. In contrast with traditional electrostatic gating, pressure increases the overlap of valence and conduction bands by moving both bands, promoting Fermi level to move in the above bands. As a result of band structure evolutions, 2H_c_‐MoS_2_ shows a gradual decrease of the indirect bandgap between the Γ point and the Q point, together with the decreased in‐plane effective mass and straight band occurrence located close to the Γ point and the K point at the Fermi level. While increasing pressure up to a quite large value, a Dirac conelike structure occurs at the Γ point. The theoretical calculations demonstrate the coexistence of two Fermi pockets near the H‐K and Γ‐A high symmetry lines at 150 GPa. It is speculated that high‐pressure‐induced dual Fermi pockets are highly connected with the occurrence of superconductivity.^[^ ^167^ ^]^ Unlike the common trigonal prismatic structure such as 2H‐MoS_2_ and 2H‐WSe_2_, WTe_2_ possesses an orthorhombic structure with a distorted octahedral coordination at ambient pressure, which implies its distinct properties at extreme pressure.^[^ ^184^, ^190^ ^]^ The superconductivity of WTe_2_ under pressure has been reported, coming from the phase transition from T_d_ to 1T′ phase.^[^ ^184^, ^190^ ^]^ This is relevant to the layer sliding of WTe_2_, which leads to a critical point of changing the interlayer spacing between Te—Te atoms. XRS experiments were conducted to verify the superconductivity (Figure 7d–f). A clear splitting of (011) and (113) peaks is found at 4–5 GPa and this is consistent with the predicted feature of superconductivity. Increasing temperature to 350 K under high pressure, the signal of the superconducting phase is stronger and dominates at higher pressure/temperature (Figure 7e,f). Further, the critical pressure of superconductivity for BP^[^ ^181^, ^194^, ^195^ ^]^ and other 2D materials^[^ ^152^, ^167^, ^186^, ^190^, ^196^, ^197^, ^198^, ^199^ ^]^ are summarized in Table 1.

### 4.5 Doping

**Figure 8:**
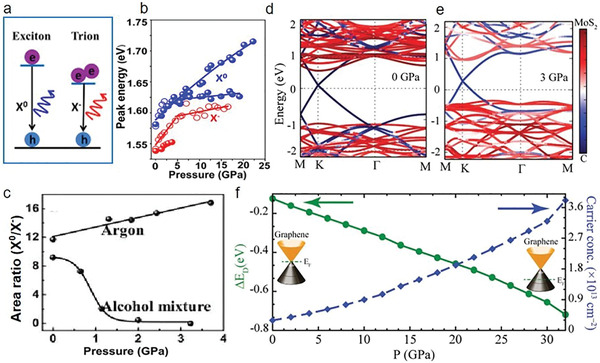
Pressure‐induced doping. a–c) Schematic diagram of the transition process for neutral exciton (X^0^) and trions (X^−^) in 2H‐MoSe_2_ monolayer, peak position evolutions of X^0^ and X^−^ versus pressure, and the PL intensity ratio of X^0^ to X^−^ as a function of pressure, respectively.^[^ ^46^ ^]^ d,e) Calculated electronic structure at ambient pressure and 3 GPa. f) The Dirac point (left axis) and the induced carrier density (right axis) of graphene from the heterostructure of graphene/MoS_2_ versus pressure. a–c) Reproduced with permission.^[^ ^46^ ^]^ Copyright 2017, American Chemical Society. d–f) Reproduced with permission.^[^ ^37^ ^]^ Copyright 2016, Wiley‐VCH.

It is imperative to modify the doping and conductivities of 2D materials for wide‐range optoelectronic applications. Lots of strategies, such as in‐plane atomic substitutions, molecular adsorption, chemical functionalization, substrate‐induced doping, etc., have been used to engineer the doping. However, most of them sacrifice the carrier mobility and conductivity or introduce spatial modulations, which limit their electronic and optoelectronic properties.^[^ ^37^, ^209^, ^210^, ^211^, ^212^, ^213^, ^214^, ^215^ ^]^ In contrast, pressure could provide a well‐controlled, stable, and impurity‐free way to engineer doping of 2D materials and corresponding heterostructures.^[^ ^37^ ^]^ As an example, fully hydrogenated graphene has been obtained under high pressure (2.6–5.0 GPa) and high‐temperature conditions, which leads to the evolution of the graphene doping level.^[^ ^216^ ^]^ After treatment under the above conditions, a sharp Raman peak at 1350 cm^−1^ (D peak) emerges. For pristine graphene, the D peak is prohibited attributed to symmetry‐based Raman selection rules. Nevertheless, it becomes dominant in the treated graphene because of the occurrence of defects or bonded atoms on the lattice that play an important role in forming sp^3^‐like sites.^[^ ^216^ ^]^ Notably, the D peak and other derivatives indicate not only the disordered or damaged graphene but also chemically tuned graphene. Importantly, these tunings are reversible by annealing the treated samples to remove hydrogen, which is impossible for structurally defective samples.^[^ ^216^, ^217^ ^]^ Moreover, the pressure‐induced charging effects and effective modulations of neutral (i.e., X^0^ coulomb‐bound electron–hole in the bandgap) and charged exciton (i.e., X^−^ two electron–hole quasiparticles in the bandgap) emissions are demonstrated in MoSe_2_ monolayers (**Figure** **8**a,b).^[^ ^46^ ^]^ In these experiments, two kinds of PTM including alcohol mixture and argon PTM were used and the corresponding pressure‐dependent energy behaviors of X^0^ and X^−^ are presented. While using the alcohol mixture PTM, X^0^ energy vanishes at 3.2 GPa and X^−^ energy firstly increases fast and then slows down from ≈5 GPa (Figure 8b). The corresponding energy evolutions could be fitted by sectional equations (i.e., *E*_g_ = 1.548 + 0.009*P* (*P* < 3.7 GPa) and *E*_g_ = 1.588 + 0.001*P* (*P* ≥ 3.7 GPa)). In contrast, using argon PTM, three peaks and X^−^ emission decrease and then disappear after 3.7 GPa. Meanwhile, after 3.7 GPa, there is a new feature located at the lower energy side whose energy evaluations could be well fitted by the equation: *E*_g_ = 1.618 + 7.365 × 10^−4^ *P*. Both this new peak and X^−^ in the alcohol mixture PTM (*P* ≥ 3.7 GPa) have a similar tendency with pressure, although there is an energy difference of ≈30 meV. More intriguingly, in these two PTM, MoSe_2_ monolayer demonstrates noncontinuous evolutions at ≈3.7 GPa that is a critical point for modulating electronic band structures. The first‐principles DFT calculations demonstrate that the conduction band K–Λ crossover occurs at this critical pressure. In alcohol mixture PTM, a suppression effect of the exciton emission and the charging effect of pressure on trions occur, which affects the oscillations of X^−^ peak and PL intensity. In argon PTM, the charging effect is blocked and thus suppression effects turn dominant. Besides, the results suggest different pressure dependences in alcohol mixture and argon PTM in terms of the ratio of the exciton to trion (X^0^/X^−^) (Figure 8c). In the former, X^0^/X^−^ ramps down rapidly with the increase of pressure and vanishes at ≈3.2 GPa. Differently, X^0^/X^−^ ramps up slightly with pressure in the latter. This supports that the alcohol mixture PTM determines the charging effect of MoSe_2_. Understandingly, with the increase of pressure, the interactions of the H atoms from the alcohol mixture PTM and Se atoms from MoSe_2_ would be stronger, leading to the emergence of the charging effect.^[^ ^46^ ^]^ Similarly, engineering the vdW interactions in heterostructures could result in the enhancement of doping.^[^ ^37^, ^218^ ^]^ The charge transfer doping of single‐layer graphene and MoS_2_ heterostructures under high pressure has been reported.^[^ ^37^ ^]^ In contrast to ambient conditions, an apparent upward movement of Dirac cone under hydrostatic pressure occurs, and this is attributed to the enhanced vdW interactions in heterostructures (Figure 8d,e). In particular, the relative shift between Dirac point and the Fermi level (*∆E*_D_) is defined to describe the charge carriers of graphene. *∆E*_D_ decreases linearly with the increase of pressure at a slope of 15.7 meV GPa^−1^ (Figure 8f) and this reveals that hydrostatic pressure could effectively modulate the carrier density of graphene. At 12 GPa, a carrier concentration of ≈1.06 × 10^13^ cm^−2^ (i.e., *∆E*_D_ of 0.38 eV) is observed, which is over 100 times higher than that of the intrinsic carrier density for graphene (≈1.06 × 10^11 ^cm^−2^) under ambient pressure. This exponential relationship between the carrier concentrations and pressure proves that pressure is an effective way to tune the carrier concentrations of monolayer graphene. Notably, 30 GPa stimulated a record‐breaking doping value of ≈3.2 × 10^13 ^cm^−2^ in a graphene/MoS_2_ heterostructure. This ultrahigh carrier concentration is ascribed to the pressure‐induced enhancement of interactions in heterostructures. Moreover, a reduction of effective distance rate (i.e., 0.06 Å GPa^−1^) between the constituent layers was observed, supporting the enhanced interlayer interactions. To explore the underlying mechanism of dopings, the planar averaged electron density difference (i.e., the total electron density of the graphene/MoS_2_ is described as *∆ρ* = *ρ*(G/MoS_2_) − *ρ*(G) − *ρ*(MoS_2_), where *ρ*(G/MoS_2_), *ρ*(G), and *ρ*(MoS_2_) represent the electron density of the graphene/MoS_2_ heterostructure, isolated graphene, and MoS_2_, respectively) was investigated. At ambient pressure, there is a charge depletion and accumulation layer in the heterostructure's interface and this is attributed to redistribution of electron density from graphene to MoS_2_. As pressure rises, the charge transfer would increase and as a result, a large upward shift of Dirac point (i.e., increasing doping) occurred. Using Bader charge transfer analysis, it demonstrates that with the increase of pressure, more charges are accumulated at the MoS_2_ layer and charge depletion occurs on the graphene side. More charge transfers from the graphene side to the MoS_2_ side with the increase of pressure. This reveals the doping tuning of graphene, which could be employed into other vdW heterostructures under high pressure. In a word, these results emphasize that pressure is a powerful tool to modulate vdW interactions, electronic structure, and doping of 2D materials and corresponding heterostructures.

### 4.6 Optical Property Tuning

**Figure 9:**
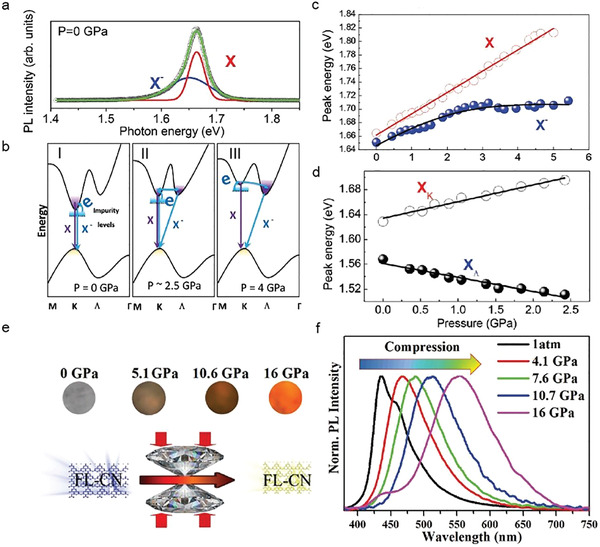
Pressure tuned optical properties. a) PL spectra of 1L WSe_2_ at ambient pressure. b) The band structure of 1L WSe_2_ at 0 GPa (I), around 2.25 GPa (II), and 4 GPa (III), respectively. c) Photon energy of X and X^−^ exciton peaks as a function of pressure in 1L WSe_2_. d) Photon energy of X and X^−^ exciton peaks as a function of pressure in 2L WSe_2_. e) Schematic illustration of PL emission color for FL‐CN samples under different pressures. f) Normalized PL spectra of FL‐CN samples at the indicated pressures. a–d) Reproduced with permission.^[^ ^221^ ^]^ Copyright 2016, Royal Society of Chemistry. e,f) Reproduced with permission.^[^ ^220^ ^]^ Copyright 2020, Royal Society of Chemistry.

Pressure also heavily tunes the optical properties of 2D materials, owing to the dramatic evolutions in band structure and interlayer interactions.^[^ ^21^, ^219^, ^220^, ^221^, ^222^ ^]^ As an example, under high pressure, the room‐temperature exciton transitions of monolayer (1L) and bilayer (2L) WSe_2_ have been explored, showing a significant evolution in direct and indirect interband transitions (**Figure** **9**a–d).^[^ ^221^ ^]^ At ambient pressure, the conduction band Λ valley of 1L WSe_2_ shows 70 ± 30 meV higher than that of K valley. The crossover of Λ–K valley occurs at around 2.25 GPa (Figure 9b). For both 1L and 2L WSe_2_, the direct K–K interband transition dominates their exciton features, which shows an apparent blueshift with the rate of 31.5 ± 0.6 (1L) and 27 ± 1 meV GPa^−1^ (2L), respectively. Correspondingly, their indirect Λ–K interband transitions demonstrate a different tendency with the increase of pressure, where the pressure coefficient is −3 ± 6 meV GPa^−1^ for 1L and −22 ± 1 meV GPa^−1^ for 2L. This illustrates that the interlayer interactions play an important role in the electronic states located on the Λ valley of WSe_2_.^[^ ^221^ ^]^ Additionally, the optical property tunings of monolayer 1H‐WS_2_ and 1H‐MoWS_2_ under high pressure have been investigated by in situ PL measurements.^[^ ^219^ ^]^ For the former, as pressure increases, the direct bandgap shows an obvious increase with a decline in PL intensity. In particular, a bandgap of 2.08 eV was obtained at 4 GPa and the PL intensity of 1H‐WS_2_ approached noise level at above 4 GPa. This hints the band transition from direct to indirect (D‐to‐I). Correspondingly, a similar tendency was observed in a 1H‐MoWS_2_ alloy in terms of both band structure and PL intensity changes, where interestingly, the critical pressure value was found at 6.1 GPa. Combining PL evolutions of MoS_2_ with pressure, it was found that the direct bandgap has a positive relationship with pressure and its definitive relations depend on their compositions.^[^ ^18^ ^]^ More intriguingly, higher W compositions induce a higher sensitivity between pressure and direct bandgap openings. The experiments demonstrate that WS_2_ has the highest rate of 54 and 36 meV GPa^−1^ for MoWS_2_. Meanwhile, the bandgap maxima before the transition of D‐to‐I was observed to be 2.05–2.08 eV despite composition variations.^[^ ^219^ ^]^ Moreover, the exciton transitions of ReS_2_ and ReSe_2_ under high pressure have been investigated experimentally and theoretically, showing a decreasing trend with the increase of pressure for both materials.^[^ ^222^ ^]^ According to photoreflectance spectroscopy and the ab initio calculations with density functional theory, the pressure coefficients of two outstanding exciton transitions are extracted. The corresponding pressure coefficients of *A* and *B* transitions for ReS_2_ is −2.3 and −4.2 meV kbar^−1^, and −3.5 and −1.3 meV kbar^−1^ for ReSe_2_. The corresponding exciton transitions are allocated to the Z *k*‐point of the Brillouin zone and the *k*‐points sitting away from high‐symmetry points. Also, these negative pressure coefficients in both materials can be interpreted by analyzing the orbitals, where pressure coefficients highly depend on the pressure‐driven destabilization of the Pz orbitals. These findings reveal a weak electronic decoupling in both ReS_2_ and ReSe_2_, which leads to the significant evolution of their optoelectronic properties in few layers as compared to that of bulks.^[^ ^222^ ^]^ Furthermore, investigations of pressure‐modified PL and bandgap evolutions of few‐layer g‐C_3_N_4_ (FL‐CN) have been conducted, exhibiting PL peak shifts from blue (434 nm) to yellow (550 nm) (Figure 9e,f).^[^ ^220^ ^]^ Besides that, an unusual PL enhancement and light absorption evolution at quite low pressures have been observed. As pressure increases, the changes of interlayer interactions play a role in photoinduced electrons and holes, which increases PL intensity. Attributed to interlayer stacking transition in FL‐CN, declining interlayer compressibility over 3 GPa occurs. This shows the smaller compressibility than that of graphite, together with prominently weakened PL intensity and broadened emission band. These results demonstrate the dominant role of interlayer interactions in optoelectronic properties of FL‐CN, contributing to an insightful understanding of its optical property tunings.^[^ ^220^ ^]^ Owing to its hybrid nature and soft lattices, the structural evolutions and optical properties of 2D organic–inorganic hybrid perovskites are quite sensitive to pressure.^[^ ^39^, ^223^ ^]^ Recently, pressure‐induced optical property evolutions of 2D phenylethylamine lead iodide perovskite crystals have been reported. At a low‐pressure range below 3.5 GPa, a continued PL redshift has been observed and this demonstrates a highly tunable energy regime up to 320 meV and unchanged quantum yield in visible spectra. Theoretical calculations illustrate that when the benzene rings in the long‐chain ligands are irradiated by the corresponding laser, the compression along the out‐of‐plane quasi‐uniaxial direction happens at high pressures. Consequently, the quantum confinement effect is tuned by 250 meV by pressure‐induced anisotropic deformations through the reduction of barrier height.^[^ ^39^ ^]^ These highly wide optical property tunings induced by pressure would enable the versatile implication of 2D materials into optoelectronic applications.^[^ ^21^, ^39^, ^219^, ^220^, ^221^, ^222^, ^223^ ^]^

### 4.7 Optimized Optoelectronic Properties and Potential Applications

High‐pressure not only enables an in‐depth understanding of the structure–property relations for 2D materials and corresponding heterostructures but also modifies their optoelectronic properties.^[^ ^14^, ^17^, ^20^, ^48^, ^167^, ^201^, ^202^, ^203^ ^]^ These high‐pressure phased properties through vdW interaction engineering extend the potential applications of 2D materials in optical, electronic, and optoelectronic fields.^[^ ^16^ ^]^ Although some optimized properties might not be retained after releasing the pressure, they are instructive for the synthesis of new 2D materials as well as designs of novel functional devices under high pressure. Also, the investigation of the alternative methods at ambient pressure may help us apply high‐pressure phased properties to 2D materials based devices. More importantly, in some cases, the high‐pressure changes could be preserved after releasing pressure. All of these reasons make high‐pressure research heavily pursued to exploit their huge potentials in optimizing properties and wide‐range practical applications.

**Figure 10:**
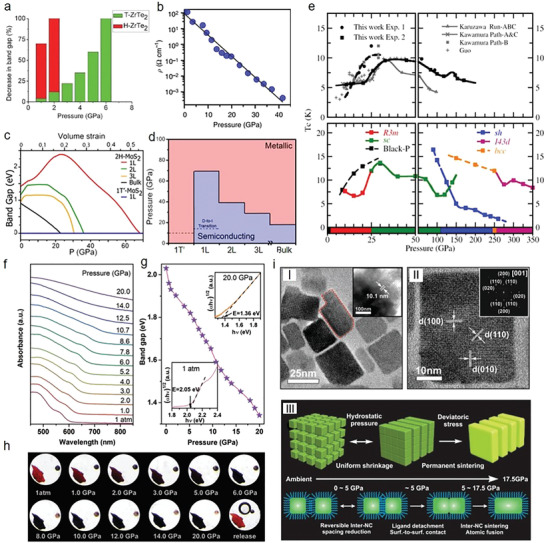
Optimized optoelectronic properties. a) The calculated bandgap change of T‐ and H‐ZrTe_2_ with the increase of pressure.^[^ ^201^ ^]^ b) Resistivity as a function of pressure for MoSe_2_. c) Bandgap changes versus pressure in various MoS_2_ polytypes. d) Pressure‐induced phase transitions in various MoS_2_ polytypes. e) Several experimental and calculated critical temperature (*T*_c_) of phosphorus under high pressure. f) Pressure‐dependent UV–vis absorption spectra of 2D Cs_3_Sb_2_I_9_ perovskite. g) The pressure‐dependent bandgap of 2D Cs_3_Sb_2_I_9_, where the inset describes the bandgap Tauc plots at 0 and 20 GPa. h) Optical images of 2D Cs_3_Sb_2_I_9_ under various pressures. i) Pressure‐induced synthesis of 2D CsPbBr_3_ perovskite nanoplates. a) Reproduced with permission.^[^ ^201^ ^]^ Copyright 2015, Royal Society of Chemistry. b) Reproduced with permission.^[^ ^20^ ^]^ Copyright 2015, Springer Nature. c,d) Reproduced with permission.^[^ ^18^ ^]^ Copyright 2014, American Chemical Society. e) Reproduced with permission.^[^ ^32^ ^]^ Copyright 2017, American Physical Society. f–h) Reproduced with permission.^[^ ^43^ ^]^ Copyright 2020, Royal Society of Chemistry. i) Reproduced with permission.^[^ ^226^ ^]^ Copyright 2017, Wiley‐VCH.

Recently, an apparent bandgap tuning of H‐ZrTe_2_ at 6 GPa was experimentally and theoretically demonstrated, which is ascribed to a transition from semiconductor to metal (**Figure** **10**a).^[^ ^201^ ^]^ Interestingly, the same transition that originates from the transverse electrical field at ambient pressure has been found as well. It is discovered that on‐state current density could be increased due to the decreasing bandgap,^[^ ^224^ ^]^ which facilities the use of Zr dichalcogenide field‐effect transistors in low‐power applications. This also implies that high‐pressure experiments provide a guide for engineering the optoelectronic properties of 2D materials in next‐generation devices. Additionally, high‐pressure‐tuned transport properties of MoSe_2_ were investigated (Figure 10b).^[^ ^20^ ^]^ From ambient pressure to 41.6 GPa, a six‐order drop in the resistivity of MoSe_2_ is observed. These changes in electronic and optoelectronic properties enable implications in energy‐variable (visible to IR) optoelectronics and photovoltaics.^[^ ^20^ ^]^ Similar with MoSe_2_, the band structure engineering could be realized in various 2D materials including MoS_2_,^[^ ^19^, ^167^ ^]^ BP,^[^ ^33^, ^181^, ^194^ ^]^ 2D perovskites,^[^ ^32^, ^40^, ^41^, ^223^, ^225^ ^]^ ZrS_2_, MoTe_2_,^[^ ^203^ ^]^ etc.^[^ ^19^, ^167^ ^]^ Moreover, pressure‐induced optoelectronic changes in MoS_2_ with varying thickness have been explored.^[^ ^18^ ^]^ In sharp contrast with bulk counterparts, 2H‐MoS_2_ monolayer's bandgap is raised by 12% due to the absence of interlayer interactions, whereas 1T′‐MoS_2_ remains in a metallic state at all pressures (Figure 10c,d). Understandingly, the vdW interlayer interactions dominate the metallization of 2D materials.^[^ ^18^, ^19^ ^]^ The interactions of sulfur atoms between the vdW gaps determine the metallizations of bilayer, trilayer, and bulk 1T′‐MoS_2_. A decreasing tendency of transition pressure is found as the layer number increases. This reversible and wide‐range tuning of bandgap provides an avenue to engineer the optoelectronic properties of 2D materials, as well as develop optimized device applications.^[^ ^18^ ^]^ How to modify the critical transition temperature (*T*_c_) of superconductivity has emerged as an important research direction since *T*_c_ is reported to be record‐breaking in sulfur hydride (SH_3_).^[^ ^199^ ^]^ Figure 10e presents several important results of the critical temperature for pressurized superconductivity in phosphorus.^[^ ^32^ ^]^ In the high‐pressure range beyond thermodynamic stability, there exist many metastable structures with larger transition temperatures as compared with putative ground‐state structures. This points out a direction to devise the materials and ameliorate their superconductivity.^[^ ^32^ ^]^ Scelta et al. unveiled the interlayer bond formations in BP at high pressure. Using Rietveld refinements, the data discloses a two‐step mechanism from the layered semimetallic rhombohedral phase (A7) to a simple‐cubic (sc) phase transition and this demonstrates the presence of an intermediate pseudo‐simple‐cubic (p‐sc) structure.^[^ ^30^ ^]^ In terms of superconductivity, BP exhibits an unusual pressure‐dependent *T*_c_ at below 30 GPa. The aforementioned p‐sc phase was illustrated by a competition mechanism between s‐p orbital mixing and electrostatic interactions. These findings provide important references in the design, synthesis, and stabilization of BP as well as relevant device applications. Moreover, unlike BP, bandgaps of 2D hybrid perovskites are too large to enable their practical application. Geng et al. reported pressure‐induced bandgap narrowing from 2.052 to 1.36 eV in 2D CS_3_Sb_2_I_9_ (Figure 10f–h).^[^ ^43^ ^]^ Both experiments and first‐principle calculations prove that the changes come from the pressurized Sb—I bond contractions and the corresponding I—Sb—I bond angle varies within [SbI_6_]^3−^ octahedral, this decides the overlap of orbitals (i.e., shifting upward the valence band and shifting downward the conduction band). These findings also unveil that the evolution of bandgap starts at ≈14 GPa and is partially reversible (e.g., the final bandgap is smaller if compared with the original one) due to an imperfect recrystallization process after pressure release.^[^ ^43^ ^]^ Likewise, the emission intensity change in 2D C(NH_2_)_3_)(CH_3_NH_3_)_2_Pb_2_I_7_ was discovered.^[^ ^31^ ^]^ PL intensity firstly increases from ambient pressure to 1.3 GPa and then decreases, finally vanishing at 7.0 GPa. Meanwhile, the bandgap of the 2D hybrid perovskite experiences a similar evolution with the PL intensity. The whole process is partially reversible, accounted by the recrystallized mechanism in perovskites. Furthermore, the recrystallization mechanism was utilized to synthesize 2D perovskite CsPbBr_3_ (Figure 10i).^[^ ^226^ ^]^ In detail, as the pressure was increased from 0 to 17.5 GPa, 2D CsPbBr_3_ experienced structural transitions, exhibiting a sequence of peak shifts and intensity changes. PL intensity shows a six times enhancement at 0.1 GPa. After pressure release, uniform and high crystalline 2D nanoplatelets were obtained due to inter‐NC fusion induced by pressure. They show a simple single‐cubic crystal structure as well as increased PL quantum yield (i.e., 1.6 times higher than that of the original sample) and longer lifetime.^[^ ^226^ ^]^ Further, we conclude the optimized optoelectronic properties and corresponding prospects for several common 2D materials in **Table** **2**. We believe that with the advancements of high‐pressure substituting methods, these high‐pressure results not only could guide the design of novel materials and high‐performance devices but also contribute to the practical applications of optimized optoelectronic properties.

**Table 2:** Optimized properties under high pressure

Materials	Optoelectronic tunings	Bandgap tuning range	Prospects
2D perovskite	Pressure‐induced amorphization during compression, PL intensity variations such as weakened PL intensity and peak position shifts, enhanced radiative excitonic recombination;^[^ ^39^ ^]^ five times PL enhancement;^[^ ^41^ ^]^ 150% PL enhancement without sacrificing the carrier lifetime;^[^ ^225^ ^]^ six times PL enhancement^[^ ^226^ ^]^	Giant tunability in bandgap including ultrabroad energy tuning of 320 meV;^[^ ^39^ ^]^ 2.05–1.36^ ^eV;^[^ ^43^ ^]^ 2.65– 2.36 eV;^[^ ^40^ ^]^ 2.00–1.92eV;^[^ ^41^ ^]^ 2.55–1.78 eV;^[^ ^223^ ^]^ 2.053–1.420 eV^[^ ^225^ ^]^	In situ optoelectronic applications or a tuning knob;^[^ ^39^, ^43^ ^]^ structure and bandgap engineering;^[^ ^40^, ^223^ ^]^ optoelectronic properties tailoring, energy applications;^[^ ^225^ ^]^ improvement in materials‐by‐design applications^[^ ^225^ ^]^
Graphene	Formation of hexagonal diamondene;^[^ ^152^, ^227^, ^228^ ^]^ giant doping of ≈6 × 10^13 ^cm^−2[^ ^229^ ^]^	Bandgap opening (e.g., trilayer graphene (2.5 ± 0.3 eV)^[^ ^230^ ^]^ and 100 meV for monolayer graphene^[^ ^229^ ^]^)	Development of carbon‐based electronic devices such as transistors or strain sensors
2D TMD	Highly tunable transport properties including the decreased resistivity or enhanced electrical conductivity;^[^ ^17^, ^20^, ^167^, ^202^, ^203^, ^231^ ^]^ enhanced onset of the critical temperature for superconductivity;^[^ ^167^ ^]^ enhanced mobility and electron concentrations as well as ionization of impurity levels;^[^ ^231^ ^]^ suppression of magnetoresistance, reconstruction of Fermi surface (the decrease of hole and increase of electron ones)^[^ ^183^ ^]^	Bandgap narrowing^[^ ^17^, ^20^, ^167^, ^201^, ^202^, ^203^ ^]^	Electronic structure and bandgap engineering; energy variable optoelectronic and photovoltaic design; alternative routing of high‐temperature superconductivity;^[^ ^167^ ^]^ optoelectronic gain modulation^[^ ^19^ ^]^
vdW heterostructures	Enhanced doping level: 0.4 × 10^13^–3.2 × 10^13 ^cm^−2^;^[^ ^37^ ^]^ enhanced charging effects in alcohol mixture PTM‐based experiments^[^ ^46^ ^]^		Tuning of electronic and band structures
MgC_2_	Enhanced electron–phonon coupling^[^ ^206^ ^]^		High temperature and ambient pressure superconductivity^[^ ^206^ ^]^
BP	Higher transition temperature of superconductivity;^[^ ^32^ ^]^ increase the pressure range of layered phased phosphorus^[^ ^30^, ^31^ ^]^ Enhanced superconducting transition temperature;^[^ ^194^ ^]^ change the dominant carrier type (a Lifshitz transition), large magnetic resistance effect, and increased effective carrier density^[^ ^181^ ^]^	Bandgap narrowing^[^ ^33^, ^181^, ^194^ ^]^	Superconducting materials design; BP and correlated materials stabilization; development of superconductivity in elemental phosphorus
Ti_3_C_2_T*_x_* MXene	Enhanced electromagnetic interference shielding performance^[^ ^232^ ^]^		Highly efficient EMI shielding applications^[^ ^232^ ^]^
h‐BN	Transformations of superhard materials phase^[^ ^26^ ^]^		Strain‐induced synthesis of superhard materials^[^ ^26^ ^]^

## 5 Our Vision: Engineering vdW Interactions in Heterostructures

When different 2D layered materials combine together via the vdW forces, vdW heterostructures form. These heterostructures not only provide a powerful platform to investigate the low‐dimensional physics but also show the huge potential for future optoelectronic and photovoltaic devices.^[^ ^129^, ^130^, ^233^, ^234^ ^]^ The vdW interactions are the key to obtain the outstanding optoelectronic performance in heterostructures, which determine their electronic band structures, charge transfer, phonon–phonon interactions, and exciton modulations (e.g., exciton quantity, energy, and dimension).^[^ ^218^, ^235^, ^236^ ^]^ Therefore, how to engineer vdW interactions decides the advancements of vdW heterostructures and corresponding functional devices.

**Figure 11:**
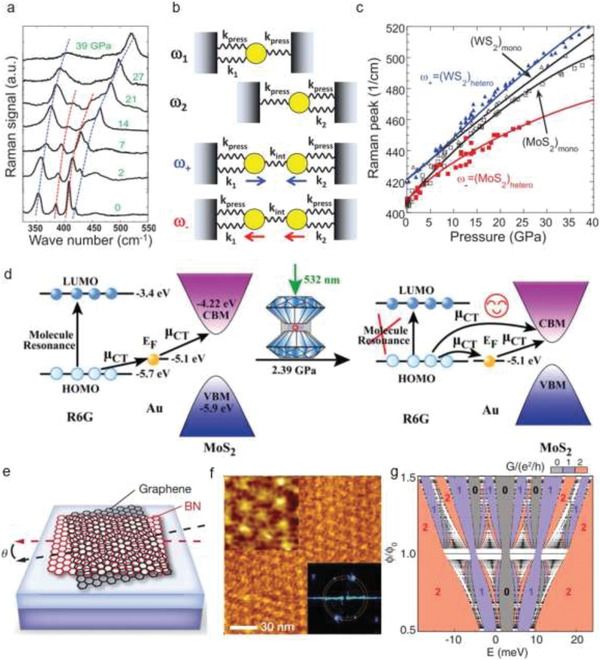
Engineering vdW interactions. a) Pressure‐dependent Raman spectra of WS_2_/MoS_2_ bilayers (the dashed lines are for guiding the eyes). b) Schematic modeling two coupled harmonic oscillators, where *ω_±_* and *k*_int_ represent the renormalized vibration frequencies and the enhanced coupling, respectively. The arrows reveal the vibration directions, which correspond to the optical‐like and acoustic‐like modes. c) The model fitting of A′_1_ modes in heterostructures [(WS_2_)_hetero_ (blue) and (MoS_2_)_hetero_ (red)]. (WS_2_)_mono_ and (MoS_2_)_mono_ stand for the A′_1_ modes of the individual TMDs. d) Surface‐enhanced Raman spectroscopy (SERS) charge transfer process in a system of MoS_2_/Au/R6G. e) Schematic of graphene stacking on h‐BN with an angle of *θ*, showing the occurrence of Moiré patterns. f) The measured Moiré patterns of graphene/h‐BN with a triangular lattice, where the upper inset demonstrates the enlarged image and the lower inset is a fast Fourier transfer of the measured regions, showing the Moiré wavelength of 15.5 ± 0.9 nm. g) Calculated Hofstadter energy spectrum of the full spin and sublattice‐spin *N* = 0 Landau level. The dense energy bands are described by the black points; the interval spectral gaps are coded with different color, which represents the corresponding two‐terminal conductance: 2 (red), 1 (purple), and 0 (gray). a–c) Reproduced with permission.^[^ ^235^ ^]^ Copyright 2015, American Physical Society. d) Reproduced with permission.^[^ ^45^ ^]^ Copyright 2015, Royal Society of Chemistry. e,f) Reproduced with permission.^[^ ^258^ ^]^ Copyright 2015, Springer Nature. g) Reproduced with permission.^[^ ^257^ ^]^ Copyright 2013, the American Association for the Advancement of Science.

Recently, the renormalizations of vibrational spectra in MoS_2_/WS_2_ heterostructures have been demonstrated at ≈39 GPa via engineering interlayer vdW interactions (**Figure** **11**a).^[^ ^235^ ^]^ In detail, in terms of the in‐plane modes (E′), both heterostructures and TMD individuals display the same tendency, which shows a linear dependence with pressure. Interestingly, their out‐of‐plane mode ($A_{1}^{{}^{\prime }}$) shows different trends. An apparent repelling phenomenon pops up where the stiffer [$A_{1}^{{}^{\prime }}$(WS_2_)_hetero_] shifts up and the softer [$A_{1}^{{}^{\prime }}$(MoS_2_)_hetero_] shifts down. The theoretical calculations reveal that $A_{1}^{{}^{\prime }}$ modes of heterostructure vibrate separately at low pressure due to the weak vdW interactions. With the increase of pressure, the interlayer vdW interactions enhance, resulting in the emergence of two coherent vibration modes for $A_{1}^{{}^{\prime }}$ modes from the constituent layers. In particular, S atoms between MoS_2_ and WS_2_ move synchronously, where one vibrates in phase, and the other vibrates along 180° out of phase. In contrast with the original $A_{1}^{{}^{\prime }}$ modes, when the two S atoms from MoS_2_ and WS_2_ layers move along the opposite (same) direction in the coherent in‐phase (out‐of‐phase) modes, the stiffened (softened) mode frequency occurs.^[^ ^235^ ^]^ Furthermore, the weakly coupled harmonic‐oscillator system is employed to explain the vibrational spectra normalization. As compared with the in‐plane lattice, if that of out‐of‐plane is much softer, pressure‐induced deformation could be simplified as the case of the uniaxial pressure along the out‐of‐plane direction. Therefore, two separate harmonic oscillators could simulate the out‐of‐plane mode of WS_2_ and MoS_2_

(1) $$\begin{array}{c} \omega_{1,2}=\sqrt{\left(k_{1,2}+2k_{\mathrm{press}}\right)/m} \end{array}$$

where *k*_1,2_ and *k*_press_ represent the intrinsic spring constant of freestanding monolayers and the increased stiffness of the spring constant for individual TMD monolayers, respectively. The latter has a positive relationship with the applied pressure. *m* represents the effective mass of two individual TMD and they are set the same for the original frequencies (*ω*_1_ and *ω*_2_). After the formation of heterostructures, the vdW interactions with spring constant *k*_int_ are added in these oscillators, the new eigenfrequencies (*ω_±_*) follow the below equation

(2) $$\begin{array}{c} \omega_{\pm }^{2}=\frac{1}{2}\left(\omega_{1}^{2}+\omega_{2}^{2}\right)+\omega_{\mathrm{int}}^{2}-\omega_{\mathrm{press}}^{2}\pm \frac{1}{2}\sqrt{{\left(\omega_{1}^{2}-\omega_{2}^{2}\right)}^{2}+4\omega_{\mathrm{int}}^{4}} \end{array}$$

where *ω*_int_ *=* $\sqrt{\;k_{\mathrm{int}}/m\;}$ and *ω*_press_ *=* $\sqrt{\;k_{\mathrm{press}}/m\;}$. In contrast with the original frequencies (*ω*_1_ and *ω*_2_), *ω*_+_ and *ω*_−_ exhibit stiffening and softening (*ω*_+_ > *ω*_1_ > *ω*_2_ > *ω*_−_), respectively (Figure 11b,c). More interestingly, the different amounts of stiffening and softening are observed, which is unlike the conventional hybridization problem (*ω*_int_ *= ω*_press_ and energy splitting is symmetric). It is found that *|ω*_+_ − *ω*_1_ *| < |ω*_2_ *− ω*_−_|, demonstrating that the weaker interaction between MoS_2_ and WS_2_ as compared with that between separate monolayers and PTM. These findings not only suggest that the vdW interactions could modify the vibration structure of layered materials but also point out a new route to explore the dimensional effects.^[^ ^235^ ^]^ Furthermore, the vdW interaction related renormalization of excitons has been reported in MoSe_2_‐WSe_2_ heterostructures.^[^ ^218^ ^]^ As pressure increases, the vdW interactions enhance and the crossing of K–Λ occurs. The 3D interlayer excitons show variations from blueshifts to redshifts with the increase of pressure and almost vanish at 2.43 GPa, where the 2D intralayer excitons are still observable. This reveals an excitonic evolution of 2D‐3D‐2D with the enhancement of the vdW interactions. Furthermore, the vdW interaction assisted lattice vibrational renormalization are found as well, where A′′_2_ modes of WSe_2_ and MoSe_2_ in heterostructures show stiffening and their out‐of‐plane modes A′_1_ show coherent behavior.^[^ ^218^ ^]^ In addition to dramatic evolutions of exciton and vibration, pressure‐induced electronic band structures modifications and two‐step charge transfer process have been demonstrated in a MoS_2_/Au/R6G system (Figure 11d).^[^ ^45^ ^]^ First, in a MoS_2_/R6G system, because the energy gap of R6G (i.e., 2.3 eV) is close to the energy of the excited laser (2.33 eV), this allows for the molecular resonance in Raman enhancements. Nonetheless, the band–band transitions between the lowest unoccupied molecular orbitals (LUMO) in R6G and VBM in MoS_2_ could not take place due to its larger transition energy (≈2.5 eV) than that of the excited laser. Pressure could effectively modulate the band structure of semiconductors, where the relation between bandgap and pressure (*E*_g_) could be described as *E*_g_ *=* 1.68 − 0.07*P* + 0.00113*P* ^2^. Consequently, as the pressure is increased to 1.64 GPa, *E*_g_ drops to 1.56 eV. Similarly, the bandgap of R6G becomes smaller, as evidenced by the redshift PL spectra. These changes make it possible for the aforementioned band–band transition (i.e., the transition between LUMO in R6G and VBM in MoS_2_). This transition would boost plenty of electrons to transfer from MoS_2_ to R6G, leading to corresponding charge transfer resonance and Raman enhancement. For the system of R6G/MoS_2_/Au, charge transfer could be divided into two steps, including electrons from highest occupied molecular orbital (HOMO) (R6G) to Au and the hot electrons from Au to the CBM in MoS_2_ (Figure 11e).^[^ ^45^, ^237^, ^238^, ^239^ ^]^ While increasing pressure from 0 to 2.39 GPa, the Au Fermi level (*E*_F_) would not change together with the upward shift of HOMO (R6G) and the downward shift of CBM (MoS_2_). This leads to the decreasing energy difference among them (i.e., *E*_F_ of Au, CBM of MoS_2_, and HOMO of R6G), promoting the charge transfer. As a result, a Raman intensity peak was observed at 2.39 GPa, where the HOMO of R6G is equal to the *E*_F_ of Au. After that, the HOMO of R6G would have higher energy than *E*_F_ of Au and their difference enlarges with the increase of pressure, which hinders charge transfer (Figure 11d). Despite several studies about engineering vdW interactions are available, the insightful understanding of vibration, charge transfer, and electronic structure changes for other heterostructures (e.g., 2D organic–organic, organic–inorganic, semiconductor–metal, etc.) under high pressure are still lacking.^[^ ^129^, ^130^, ^240^ ^]^ Moreover, versatile exciton physics (e.g., bright, dark, localized, biexciton complex, interlayer excitons, etc.) under high pressure need further investigations.^[^ ^128^, ^241^, ^242^, ^243^, ^244^, ^245^, ^246^, ^247^ ^]^ For example, how pressure tunes the spectra of dark and bright states is still unclear, which is critical to the optical response and the nonequilibrium dynamics of 2D materials under high pressure. Additionally, the pressure‐induced modifications of exciton–phonon and exciton–exciton scattering deserve a deeper understanding. Furthermore, how pressure stimulates the spin and momentum‐forbidden dark excitons affects the exploitation of potential applications for exciton‐based devices. On the other hand, when heterostructures consist of layered materials with a twist or a lattice mismatch, nanoscale Moiré patterns usually emerge due to weak vdW interactions (Figure 11e–g).^[^ ^248^, ^249^, ^250^, ^251^, ^252^ ^]^ These Moiré patterns manipulate heterostructures’ electronic and optoelectronic properties and result in multiple interesting phenomena, opening up a new direction of nano‐optoelectronics/electronics.^[^ ^248^, ^249^, ^253^, ^254^, ^255^, ^256^ ^]^ As an example, the physics of Moiré patterns in h‐BN/graphene heterostructures has been investigated, demonstrating that the periodic potential result in the formation of new Dirac cones, bandgap opening, and the emergence of Hofstadter butterfly states (Figure 11g).^[^ ^257^, ^258^, ^259^, ^260^, ^261^ ^]^ Additionally, Moiré patterns of MoSe_2_/MoS_2_ bilayers are reported, demonstrating that in‐plane potential fluctuations split the trion and exciton transitions of the constituent layers into two peaks and they correspond to the optically active local minima of Moiré potentials. Furthermore, PL dynamics reveal that the oscillator strength of transitions is insensitive to Moiré potential, which plays a key role in hindering the interlayer transfer of the thermalized excitons.^[^ ^249^ ^]^ As shown above, vdW interactions are the key to design vdW heterostructures. More importantly, besides the spatial alignments of two constituent layers, pressure provides routing to quantitatively determine the impact of vdW interactions on the electronic structure, electronic, and optoelectronic properties of heterostructures.^[^ ^55^, ^248^, ^249^, ^253^, ^254^, ^262^ ^]^

## 6 Conclusions and Outlook

In summary, we discuss pressurized optoelectronic and physics properties of 2D materials, involving structural tuning, phonon dynamics, metallization, superconducting, doping, optical property tuning, and optimized properties. The novel phenomena stimulated by pressure and underlying origins are carefully analyzed. This not only enables insight into vdW interaction engineering and the structure–property relations of 2D materials but also promotes the design and synthesis of desired properties. Moreover, we give a vision for vdW interaction engineering in heterostructures in terms of vibration, charge transfer, exciton physics, and Moiré pattern.

Nonetheless, there are still many challenges for high‐pressure research of 2D materials and heterostructures. 1) More and higher‐resolution characterization methods are required to deepen the understanding of the vdW interaction engineering and structure–property relations under high pressure; 2) The sample in DAC is small and not nonuniform, which leads to high uncertainty and poor reproducibility of results; 3) Ultrathin 2D materials including BP, 2D perovskites, and some of TMDs show poor chemical stability and interact actively with air, moisture, and laser illumination. This leads to unrecoverable degradation that affects the reliability of the results under high pressure. 4) It is difficult to achieve an ultrahigh pressure of over 400 GPa, which is commonly used to probe novel materials’ properties under extreme conditions. 5) Theoretical simulations and modeling are immature to fully interpret experimental findings (e.g., how to explain the pressurized assembly process). Given these, we believe that a variety of exciting investigations and applications on 2D materials under high‐pressure will emerge in the coming future.

## Conflict of Interest

The authors declare no conflict of interest.
